# TamL is a Key Player of the Outer Membrane Homeostasis in Bacteroidota

**DOI:** 10.1016/j.jmb.2025.169063

**Published:** 2025-03-03

**Authors:** Fabio Giovannercole, Tom De Smet, Miguel Ángel Vences-Guzmán, Frédéric Lauber, Rémy Dugauquier, Marc Dieu, Laura Lizen, Jonas Dehairs, Gipsi Lima-Mendez, Ziqiang Guan, Christian Sohlenkamp, Francesco Renzi

**Affiliations:** 1- **Research Unit in Biology of Microorganisms (URBM),** Namur Research Institute for life Sciences (Narilis), University of Namur, Namur, Belgium; 2- **Centro de Ciencias Genómicas,** Universidad Nacional Autónoma de México, Av. Universidad s/n Col. Chamilpa, C.P. 62210 Cuernavaca, Morelos, Mexico; 3- **Technological Platform Mass Spectrometry Service (MaSUN),** Namur Research Institute for Life Sciences (Narilis), University of Namur, Namur, Belgium; 4- **Laboratory of Lipid Metabolism and Cancer,** Department of Oncology, KU Leuven, Leuven, Belgium; 5- **Department of Biochemistry,** Duke University School of Medicine, Durham, NC 27710, United States

**Keywords:** translocation and assembly module (TAM), Bacteroidota, outer membrane vesicles, envelope homeostasis, bacterial membranes

## Abstract

In Proteobacteria, the outer membrane protein TamA and the inner membrane-anchored protein TamB form the Translocation and Assembly Module (TAM) complex, which facilitates the transport of autotransporters, virulence factors, and likely lipids across the two membranes. In Bacteroidota, TamA is replaced by TamL, a TamA-like lipoprotein with a lipid modification at its N-terminus that likely anchors it to the outer membrane. This structural difference suggests that TamL may have a distinct function compared to TamA. However, the role of TAM in bacterial phyla other than Proteobacteria remains unexplored. Our study aimed to elucidate the function of TamL in *Flavobacterium johnsoniae*, an environmental Bacteroidota. Unlike its homologs in Proteobacteria, we found that TamL and TamB are essential in *F. johnsoniae*. Through genetic, phenotypic, proteomic, and lipidomic analyses, we show that TamL depletion severely compromises outer membrane integrity, as evidenced by reduced cell viability, altered cell shape, increased susceptibility to membrane-disrupting agents, and elevated levels of outer membrane lipoproteins. Notably, we did not observe an overall decrease in the levels of β-barrel outer membrane proteins, nor substantial alterations in outer membrane lipid composition. By pull-down assays, we found TamL co-purifying with TamB in *F. johnsoniae*, suggesting an interaction. Furthermore, we found that while TamL and TamB monocistronic genes are conserved among Bacteroidota, only some species encode multiple TamL, TamB and TamA proteins. To our knowledge, this study is the first to provide functional insights into a TAM subunit beyond Proteobacteria.

## Introduction

All Gram-negative (diderm) bacteria possess an outer membrane (OM) surrounding the periplasm, the aqueous compartment that separates the OM from the inner membrane (IM).^1^ In most diderm bacteria, the OM is made of phospholipids (PLs) and lipopolysaccharide (LPS), whose distribution is asymmetrical along the OM, with PLs and LPS located in the inner and outer leaflets, respectively.^1^ Additionally, the OM is enriched with proteins, such as integral OM proteins (OMPs) that adopt a characteristic β-barrel fold, and lipoproteins, which are globular proteins anchored to the OM via a lipid moiety.^2^ Both OMPs and OM lipoproteins are synthesized in the cytoplasm as precursors and are then translocated across the periplasm towards the OM thanks to dedicated shuttle systems, such as the periplasmic protein LolA in the case of OM lipoproteins or the periplasmic chaperones Skp, SurA and DegP for OMPs.^3^ After reaching the OM, the insertion and folding of OMPs in the OM is catalyzed by BamA, a members of the Omp85 family,^4,5^ whereas OM lipoproteins are inserted in the OM by LolB.^2,3^

The OMP BamA is the best characterized member of the Omp85 family and the main subunit of the BAM (β-Barrel Assembly Machinery) complex, which assembles OMPs in all diderm bacteria.^6-9^ However, some components of a family of outer membrane/secreted proteins known as autotransporters seem to rely on an auxiliary complex, called Translocation and Assembly Module (TAM).^10-13^ TAM is composed of TamA, a BamA homolog, which is the OM foldase and insertase of the complex, and of an IM-anchored periplasmic protein TamB, which might convey nascent and unfolded OMPs from the IM to TamA.^14,15^ The full-length structure of TamA has been determined^16,17^ and, like other members of the Omp85 family, it contains three N-terminal POlypeptide TRansport Associated (POTRA) domains and a C-terminal β-barrel domain.^15,16^ As for TamB, only the structure of a limited portion (residues 977–1136) of the *Escherichia coli* TamB has been experimentally solved, which folds into a hydrophobic β-taco motif consisting of β-sheets and random coils.^18^ Predictions indicate that TamB periplasmic portion likely consists of repetitions of this fold, thus forming a tube-like structure, which may assist in chaperoning OMPs to TamA.^10,11^ Furthermore, the fold adopted by the last six β-strands of the C-terminal end of TamB mirrors that of OMP β-barrels, suggesting a pseudosubstrate function.^10,15^ This pseudosubstrate domain likely interacts with the β-barrel domain of TamA until high affinity substrates bind.^13,19^

TamB belongs to a family known as AsmA-like proteins, which in *E. coli* includes five additional members, AsmA, YicH, YhjG, YhdP and YdbH, all anchored to the IM.^20^ More recently, seven AsmA-like proteins were also identified in *Pseudomonas aeruginosa*.^21^ These AsmA-like proteins have in common the periplasmic β-taco fold, and thereby their tube-like structures resemble that of the eukaryotic repeated β-groove (RBG) lipid-transfer proteins.^22-24^ Recent experimental evidence has shown that in *E. coli* YhdP, YdbH and TamB are synthetically lethal. In fact, the absence of YhdP and TamB leads to cell lysis, increased antibiotic susceptibility, envelope defects and LPS excess shed via outer membrane vesicles (OMVs) likely owing to a reduced anterograde (IM to OM) transport of GPLs, which were found to accumulate in the IM.^19,25-29^

To date, the structure and function of TAM have been investigated in depth exclusively in Proteobacteria, where the genes coding for TamA and TamB are part of the same operon. However, unlike TamA, TamB is ubiquitously distributed across almost all diderms, which suggests that it may have other functions compared to its proteobacterial homologs.^9,30^ For instance, in the Spirochaetota (previously known as Spirochetes) *Borrelia burgdorferi*, which lacks TamA, TamB is essential and interacts with BamA.^31^ Therefore, what is currently known about TAM in Proteobacteria could not necessarily be extended to members of other phyla.

Members of the Bacteroidota and Chlorobiota phyla (previously known as Bacteroidetes and Chlorobi, respectively) lack TamA, which is instead replaced by TamL, a TamA-like lipoprotein.^9,30^ In fact, TamL proteins possess a characteristic lipoprotein signal peptide harboring a conserved cysteine to which lipid moieties are attached during protein maturation. This suggests that, unlike TamA, the TamL periplasmic POTRA domains are likely anchored to the OM.^9^ The reason for this difference between TamL and TamA is unknown. Furthermore, it must be pointed out that in general the OM lipid and protein composition in Bacteroidota significantly differs from that of Proteobacteria.^32-36^ Thus, these biochemical features raise questions about TamL function and its ability to interact with TamB in Bacteroidota.

In this work we aimed to investigate the biological role of TamL in OM homeostasis and biogenesis of the Bacteroidota *Flavobacterium johnsoniae*, an environmental and model bacterium for gliding and Type 9 secretion system (T9SS).^37-39^ We show that in *F. johnsoniae* TamL is essential, and that its depletion drastically affects OM integrity, induces blebs with release of large OMVs, and drastically perturbs the OM proteome composition, while having only a minor impact on the OM lipidome. Additionally, we show that TamL copurifies with TamB, suggesting an interaction. We also confirmed the essentiality of TamL, as well as that of TamB, in the Bacteroidota *Capnocytophaga canimorsus*, an oral commensal of cats and dogs and a human pathogen.^33,40,41^ Finally, we found that *tamL* and *tamB* genes in Bacteroidota are not part of the same operon, a genetic feature that appears to be exclusive to members of this phylum.

## Results

### F. johnsoniae possesses several TamL and TamB homologs, some of which are essential

**Figure 1.**
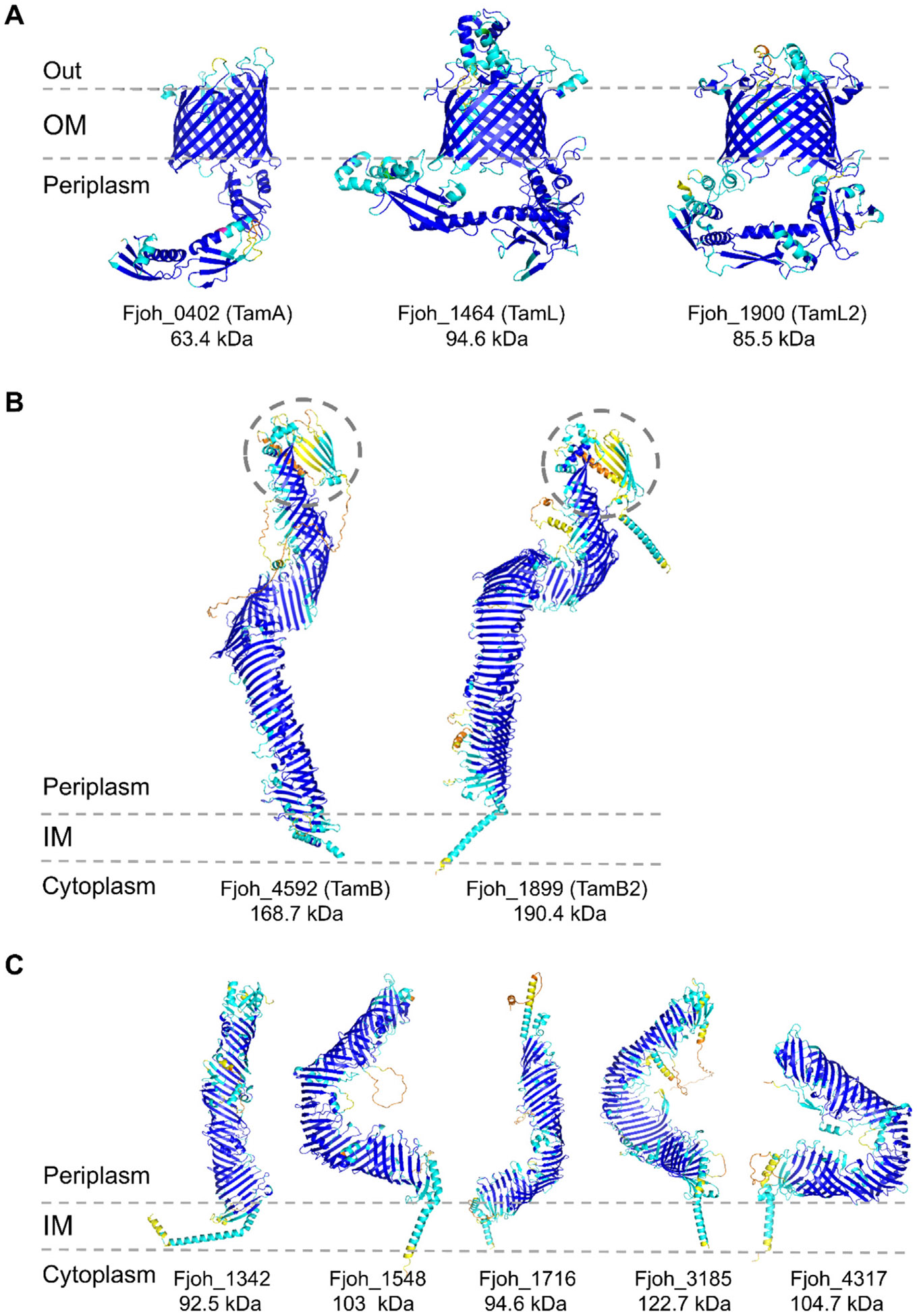
Three-dimensional structures of TamA (A), TamB (B) and AsmA-like (C) homologs in *F. johnsoniae* as predicted by AlphaFold2 (https://alphafold.ebi.ac.uk).^86^ Protein names and their predicted molecular weights are reported below each structure. Structures are colored based on the per-residue confidence score (pLDDT) between 0 and 100: dark blue (pLDDT > 90), cyan (90 > pLDDT > 70), yellow (70 > pLDDT > 50), and orange (pLDDT < 50). In (A), the N-terminal lipid moiety that likely anchors TamL and TamL2 to the OM is not shown. In (B), the predicted C-terminal pseudosubstrate domain^10^ is highlighted via a dashed circle.

We first searched for a putative TamL homolog in *F. johnsoniae* by DELTA-BLAST^42^ using TamA from *E. coli* (*Ec*TamA, UniProt code: P0ADE4) as a query. We identified three proteins annotated as Fjoh_1900, Fjoh_1464 and Fjoh_0402 (Table S1). Their AlphaFold2-predicted structures revealed the presence of three N-terminal POTRA domains and of a C-terminal β-barrel made of 16 β-sheets, alike *Ec*TamA (Figure 1A). Interestingly, Fjoh_1900 and Fjoh_1464 were predicted with high likelihood (0.99 and >0.83, respectively) to have a lipoprotein signal peptide (cleaved by signal peptidase II, SPase II)^43^ preceding a cysteine residue, Cys27 (Fjoh_1900) and Cys21 (Fjoh_1464), which is the predicted lipidation site. As a result, we concluded that both Fjoh_1464 and Fjoh_1900 are TamL homologs, and we named them TamL and TamL2, respectively. In contrast, a SPI signal peptide (cleaved by signal peptidase I, SPase I)^43^ was observed (likelihood > 0.99) for Fjoh_0402, thus indicating that Fjoh_0402 is not a lipoprotein, alike *Ec*TamA. We therefore concluded that Fjoh_0402 is a TamA homolog in *F. johnsoniae*. This finding was unexpected since it was reported that TamA is nearly exclusively confined to Proteobacteria, where its gene (*tamA*) is in operon with *tamB*.^9,30^ Conversely, we did not identify any TamB-encoding gene flanking *fjoh_0402*.

We then sought putative TamB homologs using *Ec*TamB as a query. We identified two proteins, Fjoh_1899 and Fjoh_4592 (Table S1). Their AlphaFold2-based predicted structures confirmed the presence of repeated units of the β-taco fold, and of the pseudosubstrate domain (residues 1321–1420 in Fjoh_4592; residues 1538–1643 in Fjoh_1899) located at the C-terminus,^18^ akin to what was already observed in EcTamB (Figure 1B).^10,30^ Interestingly, the Fjoh_1899 encoding gene (*fjoh_1899*) may be in operon with *fjoh_1900* (*tamL2*) since the start codon of the latter precedes the stop codon of the former. We therefore named Fjoh_4592 and Fjoh_1899 TamB and TamB2, respectively. Notably, the C-terminal end of TamB (residues 1416–1494), but not of TamB2, is portrayed as disordered in the AlphaFold model (Figure 1B).

We also sought additional AsmA-like proteins in *F. johnsoniae* using the *E. coli* AsmA protein as a query. Interestingly, in addition to TamB2, we identified five proteins: Fjoh_1342, Fjoh_1548, Fjoh_1716, Fjoh_3185 and Fjoh_4317 (Table S1). Their AlphaFold2-based predictions strongly support their identity as AsmA-like proteins (Figure 1C). As a result, we concluded that *F. johnsoniae* possesses in total seven AsmA-like proteins.

To investigate the role of the TamL and TamB homologs in *F. johnsoniae*, we deleted their encoding genes. Surprisingly, while we could delete *tamA*, *tamB2* and *tamL2*, as well as codelete *tamB2* and *tamL2*, no deletion mutants could be obtained for *tamB* and *tamL*. Furthermore, attempts to generate a TamL variant in which the predicted site of lipidation was mutated (Fjoh_1464-C^21^G) were unsuccessful. This suggests that both *tamL* and *tamB* are essential genes, and that the N-terminal lipidation of TamL is crucial. Additionally, mutants lacking *tamA*, *tamB2*, or *tamL2* grew to the same extent as the wild-type (WT) strain in rich (CYE) and motility media (MM), suggesting no major effect of their deletion on cell fitness under these conditions (Figure S1). Therefore, we decided to restrict our study to TamB and TamL.

### TamB and TamL have distinct regulation within the cell

**Figure 2.**
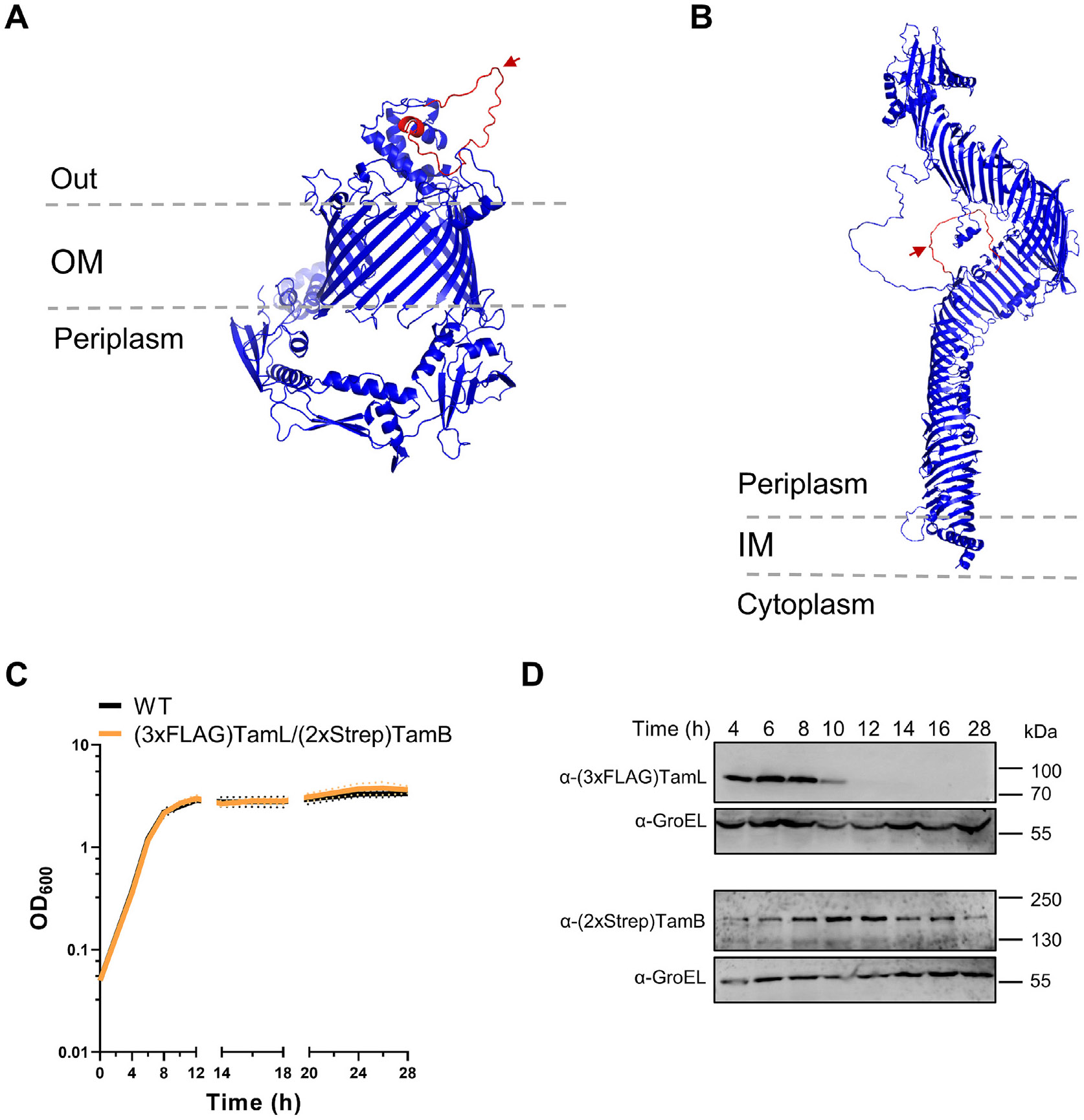
TamL and TamB expression pattern throughout cell growth. Structural models of the tagged variants of TamL (A) and TamB (B) as predicted by AlphaFold2.^84,86^ The 3xFLAG-tag sequence in TamL (A) and the 2xStrep-tag sequence in TamB (B) are highlighted in red and indicated by red arrows. In (A), the N-terminal lipid moiety that likely anchors TamL to the OM is not displayed. (C) Growth curves in CYE of *F. johnsoniae* WT (black) and (3xFLAG)TamL/(2xStrep)TamB (orange) strains showing no significant effect of the two tags on cell growth. Data are displayed as mean ± standard deviation from at least three biological replicates. (D) TamL and TamB immunodetection throughout cell growth. GroEL detection was used as a loading control.

In Proteobacteria, *tamA* and *tamB* are part of the same operon, suggesting that TamA and TamB likely undergo the same spatial–temporal regulation in order to form an active TAM complex.^9,30^ To test if the same could occur in *F. johnsoniae*, we generated strains expressing tagged variants of TamL and TamB, where a tag sequence was inserted into the genomic loci of *tamL* and *tamB*. In the case of TamL, we inserted a 3xFLAG tag, flanked by four glycine residues on both extremities, in a non-conserved loop of TamL (^574^TNQV^577^) predicted to be extracellular (Figure 2A). As for TamB, we added a Twin-strep (2xStrep) tag at the C-terminal end (Figure 2B). The 3xFLAG-TamL/2xStrep-TamB double-tagged strain exhibited no significant growth difference compared to the WT strain when grown in CYE medium (Figure 2C), which indicated that the two tags do not have an impact on TamL and TamB function. We then monitored the expression of both proteins over time and we found that, while TamB signal was detectable throughout all time points with a slight increase during the transition from exponential to stationary phase of growth, TamL detection was limited to the initial eight hours of growth spanning from early to late-exponential phase (Figure 2C and D). This may indicate that TamL, but not TamB, likely undergoes strict regulation at the protein level. As a result, TamL and TamB are both present only when cells are actively growing.

### TamL depletion causes loss of cell viability and leads to shape abnormalities

**Figure 3.**
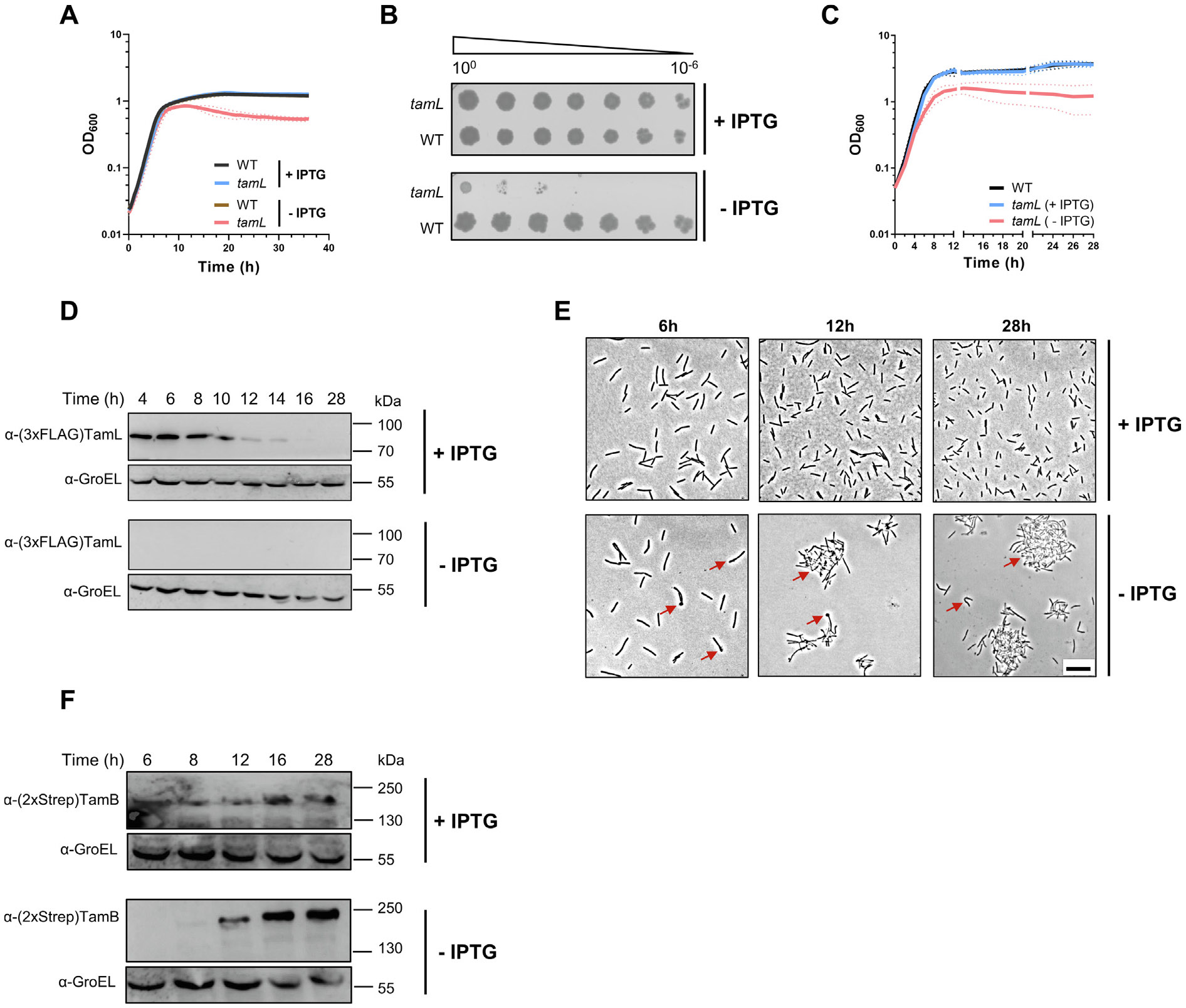
TamL depletion causes loss of cell viability and shape abnormalities. (A) Growth curves of *F. johnsoniae* WT and P_ompA_::*lacI*-P_cfxA-lacO_::*tamL* (*tamL*) strains in CYE (±IPTG). (B) Spot assay of cells grown in CYE (±IPTG). Plates were incubated at 30 °C and photographed after 48 h. (C) Growth curves in flasks (25 ml) of *F. johnsoniae* WT (black) and P_ompA_::*lacI*-P_cfxA-lacO_::*tamL* (*tamL*) strains in CYE in permissive (+IPTG, blue) and non-permissive (−IPTG, red) conditions. (D) Immunoblotting of (3xFLAG)TamL. GroEL signal was used as loading control. (E) Imaging of cells at different time points grown in CYE (±IPTG). OD_600_-normalized cells were fixed in 4% paraformaldehyde, and then visualized by phase-contrast microscopy. Red arrows indicate cell shape abnormalities and aggregation. Scale bar represents 10 μm. (F) Immunoblotting of (2xStrep)TamB. Data in (A) are displayed as mean ± standard deviation from three independent experiments.

Given the essentiality of *tamL* and *tamB* in *F. johnsoniae*, we aimed to knock-down their expression by replacing their native promoters with the IPTG-inducible P_cfxA-lacO_ promoter. While we succeeded in generating the *Fjoh* P_ompA_::*lacI*-P_cfxA-lacO_::*tamL* strain in the (3xFLAG)*tamL/*(2xStrep)*tamB* genetic background, we could not replace the native *tamB* promoter despite several attempts and different strategies (see materials and methods for more details). Therefore, we restricted our study to *tamL*. We cultured the *Fjoh* P_ompA_::*lacI*-P_cfxA-lacO_::*tamL* strain in CYE medium with or without IPTG (±IPTG), corresponding to permissive or non-permissive conditions of growth, respectively. We noticed that, while in permissive conditions the growth of *Fjoh* P_ompA_::*lacI*-P_cfxA-lacO_::*tamL* mirrored that of the WT strain, in non-permissive conditions its growth curve exhibited a decline starting approximately at ten hours when grown on a 96-well plate (Figure 3A). Likewise, we observed a significant growth reduction on plate (Figure 3B). To confirm that the observed growth defects were caused by TamL depletion, we monitored TamL abundance over time during growth in flasks (Figure 3C and D). In this condition, the growth in non-permissive conditions was significantly affected already after six hours, followed by a growth decline after 12–14 h (Figure 3C). As for TamL abundance, in permissive conditions, even if expressed constitutively, TamL expression profile mirrored that of the WT strain (Figure 3C and D), thus suggesting a post-translational regulation. In contrast, in non-permissive conditions, we could not detect TamL (Figure 3D). Furthermore, by phase-contrast microscopy we noticed that cells grown in non-permissive conditions displayed severe morphological defects unlike those grown with IPTG. Already after six hours of growth (Figure 3C and E), approximately 15–20% of the cells exhibited a lollipop-like shape, which were then replaced by more severe shape irregularities that became prominent over time, as indicated by the accumulation of a substantial cluster of cells and debris at later time intervals (12 h and 28 h, Figure 3C and E). These results indicate that the growth of *Fjoh* P_ompA_::*lacI*-P_cfxA-lacO_::*tamL* is IPTG-dependent, and that TamL depletion is the cause of the observed phenotypes in non-permissive conditions.

Interestingly, we noticed a sharp increase in TamB protein levels over time in non-permissive conditions (Figure 3C and F). Conversely, we found that the deletion of *tamA* and *tamL2* did not significantly aggravate cell fitness in TamL-depleted cells, suggesting that neither of them can substitute for TamL (Figure S2).

### TamL depletion disrupts outer membrane integrity, with increased release of OMVs

**Figure 4.**
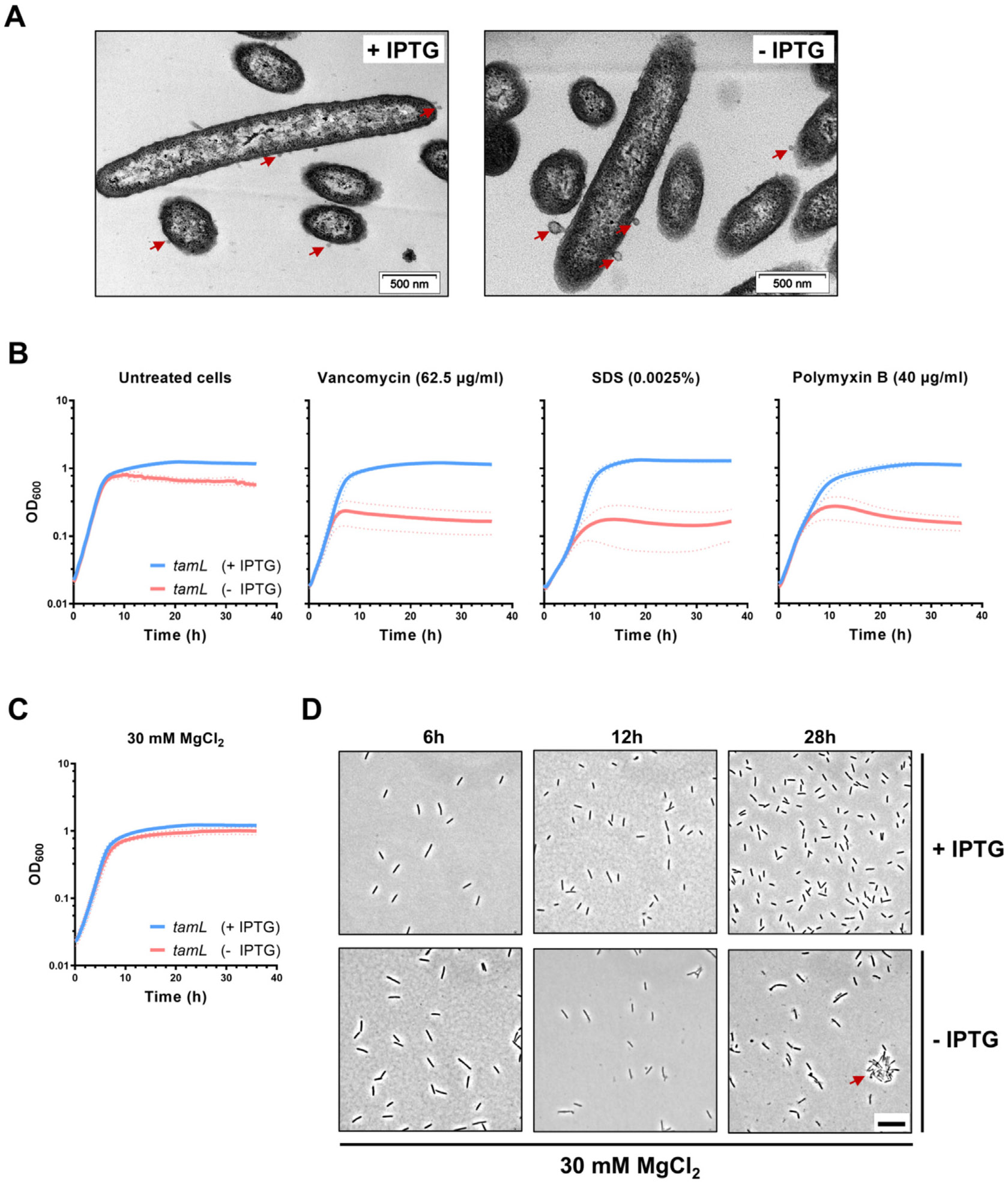
TamL depletion results in increased OM permeability and release of bigger OMVs. (A) TEM imaging of cells grown in ± IPTG. Representative OMVs are indicated by red arrows. (B and C) Growth curves of *F. johnsoniae* P_ompA_::*lacI*-P_cfxA-lacO_::*tamL* (*tamL*) in CYE (±IPTG). (B) Cells were incubated without (untreated cells) or with the envelope perturbing agents at the indicated concentrations. (C) Cells were grown with increased concentration of Mg^2+^. (D) The increment in Mg^2+^ partially rescues the envelope defects in TamL-depleted cells. Cells were grown in CYE (±IPTG) supplemented with 30 mM MgCl_2_. At different time points, OD_600_-normalized cells were collected, fixed in 4% paraformaldehyde, and then visualized by phase-contrast microscopy. The red arrow at the bottom right panel indicates cell aggregation. Scale bar represents 10 μm.

Intrigued by the morphological defects observed in TamL-depleted cells, we looked at cells grown in permissive or non-permissive conditions by transmission electron microscopy (TEM) to detect shape abnormalities at higher resolution. While in both growth conditions we could detect the presence of spherical structures resembling OMVs, in non-permissive conditions the OMVs appeared bigger (Figure 4A, see also Figure 6B for diameter quantification). Prompted by this finding, we grew cells in the presence of several OM perturbing agents (i.e., Polymyxin B, Vancomycin and SDS) to assess if the depletion of TamL determined an increased susceptibility to them. As shown in Figure 4B, cells incubated in the presence of the three envelope perturbing agents displayed a strong reduction in cell viability compared to the control condition (untreated cells) only when grown in non-permissive conditions, whereas a negligible effect was observed in permissive conditions at the tested concentrations.

In *E. coli*, it was shown that loss of OM material in mlaA* cells (i.e., cells where phospholipids are pushed from the inner to the outer leaflet of the OM with concomitant increase in phospholipid transport from the IM to the OM), and the lysis observed in the *ΔyhdPΔtamB* mutant could be both partially reduced by adding Mg^2+^ in the medium.^25,44,45^ Accordingly, when we increased the concentration of Mg^2+^ (up to 30 mM) in CYE we observed that, in non-permissive conditions, it partially ameliorated cell growth (Figure 4C) and reduced but not abrogated shape abnormalities (Figure 4D).

Overall we concluded that TamL depletion in *F. johnsoniae* is detrimental for cell viability and is accompanied by severe membrane and permeability defects, suggesting that TamL is required for OM integrity and homeostasis.

### TamL depletion alters outer membrane (lipo) protein composition

**Figure 5.**
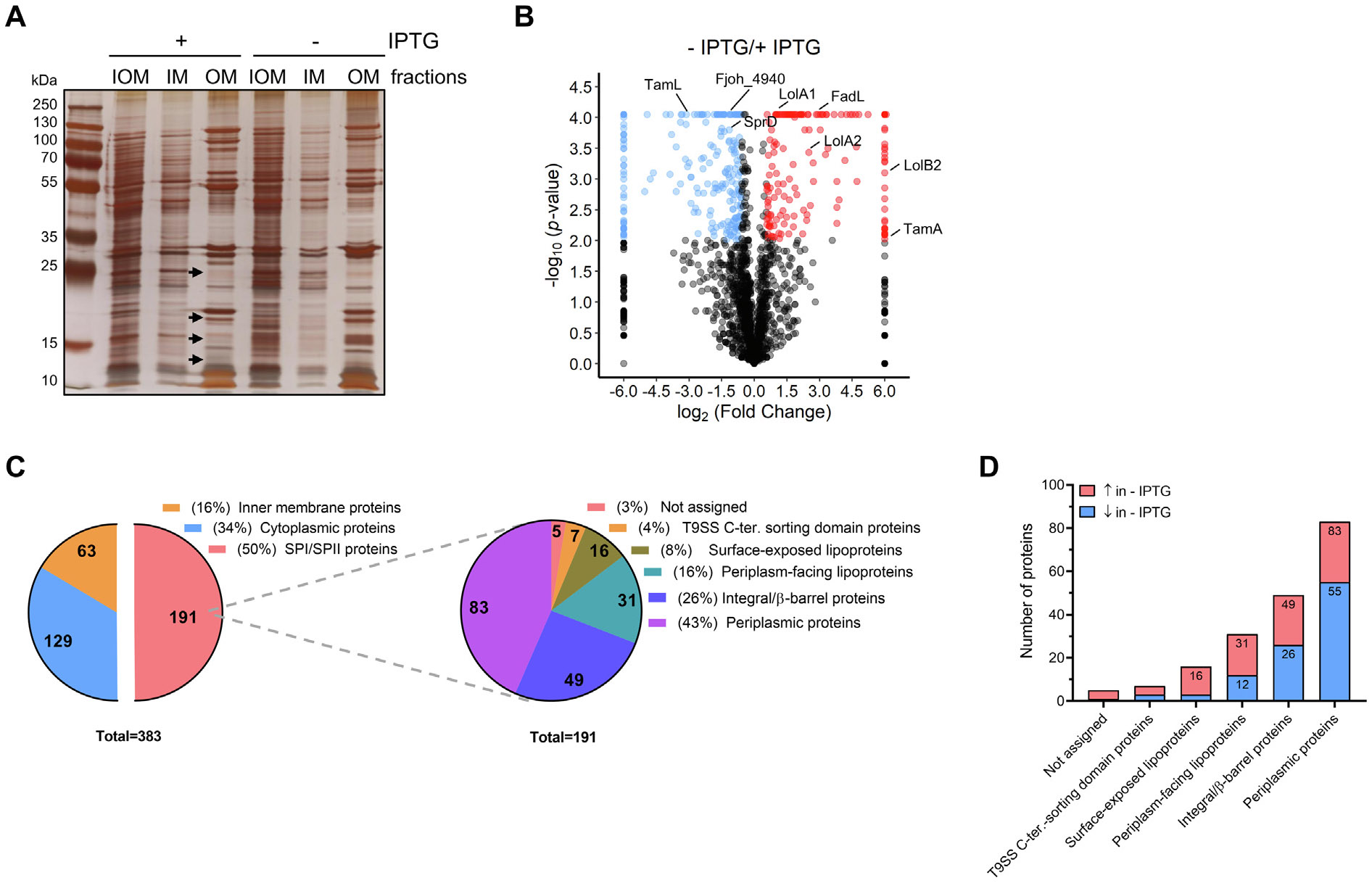
TamL depletion significantly affects the OM protein composition. (A) AgNO_3_–stained polyacrylamide gel (12%) of total membrane (IOM), inner membrane (IM) and outer membrane (OM) fractions isolated from cells grown in CYE (±IPTG). Black arrows indicate protein bands of the OM fractions showing different intensities in the two conditions (±IPTG). (B) Volcano plot depicting the fold change (FC) and the statistical significance (*p*-value) of the proteome of the OM fractions (±IPTG). The FC corresponds to −IPTG/+IPTG ratio. Blue and red dots indicate significantly decreased or increased proteins (FC 𢉥 ∣1.5∣; *p* < 0.01), respectively. Black dots represent those proteins either not significantly affected (FC < ∣1.5∣) or with a *p*-value ≥ 0.01. The projection of some proteins described in the main text is shown. (C) Left: pie chart showing the number of IM proteins, cytoplasmic proteins and proteins with a signal peptide (SPI/SPII) and their relative abundance (%) over the total number of significantly affected proteins. Right: same as left but depicting the number and the relative abundance of the different classes of SPI/SPII proteins over the total number of significantly affected SPI/SPII proteins. (D) Bar chart showing the number of different classes of SPI/SPII proteins found significantly increased (red) or decreased (blue) upon TamL depletion.

The above results clearly support the key function of TamL in maintaining OM integrity. Hence, we wondered if the observed phenotypes could be attributed to an alteration of the OM proteome caused by TamL depletion. We isolated the membrane fractions from cells grown in permissive and non-permissive conditions by ultracentrifugation and treatment with the ionic detergent Sarkosyl, which selectively solubilizes the IM but not the OM (see materials and methods and Figure S3). While the total protein content (i.e., total protein mass in mg) in each fraction was similar between conditions (Figure S4), SDS-PAGE analysis indicated a clear difference in protein composition (Figure 5A).The mass spectrometry (MS) analysis identified 383 proteins significantly affected (FC ∥ ∣1.5∣; *p* < 0.01) (Figure 5B), with 191 (50%) predicted to have a signal peptide (SPI or SPII), thus localizing in the periplasm and/or the OM (Figure 5C and Table S2). Moreover, 129 (34%) of these proteins were predicted as cytoplasmic and 63 (16%) as IM proteins (Figure 5C and Table S3). Despite the presence of cytoplasmic and IM proteins, the analysis of protein abundance (i.e., number of identified peptides *per* protein) revealed that SPI/SPII proteins were the most abundant in all OM samples (Figure S5 and Table S4), confirming the effectiveness of the purification.

Of the 191 significantly affected SPI/SPII proteins, 91 (48%) were increased and 100 (52%) were decreased upon TamL depletion (Table S2). The most abundant proteins were periplasmic (83), followed by integral/β-barrel proteins (49) (i.e., proteins anchored to the OM as part of a complex or embedded into the OM due to the β-barrel domain), OM lipoproteins (47), and proteins with a Type 9 secretion system (T9SS) C-terminal sorting domain (7).^1,38,39,46^ The localization could not be assigned for five proteins. Notably, 16 of the 47 OM lipoproteins were predicted to be surface-exposed (i.e., anchored on the outer leaflet of the OM), owing to the presence of the lipoprotein export signal (LES) typical of members of the Bacteroidota phylum^34^; in contrast, the remaining 31 were predicted to be anchored to the OM inner leaflet, and thereby facing the periplasm (periplasmic OM lipoproteins).

Although TamL depletion did not result in a global increase or decrease in integral/β-barrel proteins, a more detailed analysis of this protein class revealed significant alterations (Table S2). For instance, we observed a substantial increase in the levels of Fjoh_4941 (FC: 5.6), which is likely a FadL homolog, as suggested by the AlphaFold2-predicted structure, PFAM annotation and DELTA-BLAST sequence alignment (E-value: 8e–85). In *E. coli*, FadL functions as a long-chain fatty acid (LCFA) transporter and plays a critical role in maintaining OM integrity by importing LCFAs for membrane lipid synthesis.^47^ Interestingly, we also found that Fjoh_0402, the TamA homolog of *F. johnsoniae*, was exclusively detected in the membrane fractions of TamL-depleted cells.

Consistent with the depletion experiments, TamL (Fjoh_1464) was one of the most significantly decreased proteins (FC: 0.1). Moreover, the amount of SprD (Fjoh_0980), part of the T9SS, was also reduced (FC: 0.4) upon TamL depletion.^46,48^ In contrast to the integral/β-barrel proteins, the majority of the OM lipoproteins (31 out of 47) were significantly upregulated, with surface-exposed lipoproteins being particularly affected (13 out of 16 with increased abundance) (Table S2). Among the periplasmic OM lipoproteins, the LolB homolog Fjoh_1084 (LolB2)^49^ showed an increase upon TamL depletion (FC: INF). Additionally, the amount of LolA homologs Fjoh_2111 (LolA1) and Fjoh_1085 (LolA2) was also increased, suggesting an upregulation of the Lol system(s) in response to TamL depletion.^49,50^ We also observed an increased abundance of the lipoprotein Fjoh_4940 (FC: 2.6) which, given the genomic proximity of its encoding gene (*fjoh_4940*) with that of the putative FadL homolog of *F. johnsoniae* (*fjoh_4941*), might indicate a functional link.

Not much information could be inferred from the OM lipoproteins decreased upon TamL depletion (16 out of 47).

Overall, from the above proteomic analysis we concluded that the depletion of TamL, while broadly affecting the OM protein content, is accompanied by a significant increase in OM lipoproteins.

### TamL depletion causes the release of large outer membrane vesicles (OMVs) with an altered protein content

**Figure 6.**
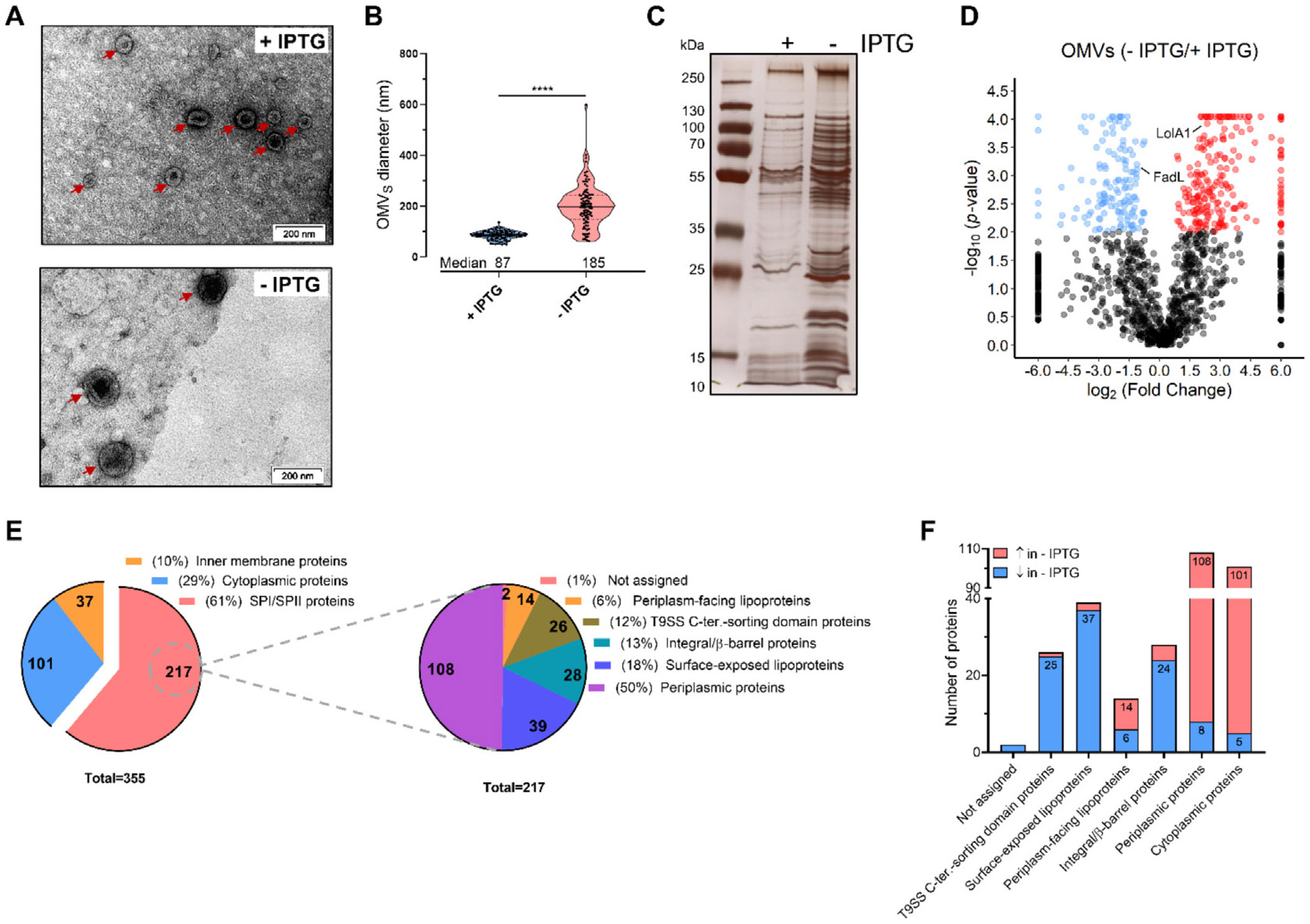
The OMVs shed from TamL-depleted cells are larger and enriched with periplasmic proteins. (A) Representative TEM images of OMVs (indicated by red arrows) from cells grown in ±IPTG. OMVs were fixed and stained before TEM visualization. (B) Violin plots quantifying the OMVs diameter (in nm). From each TEM image, the diameter of the OMVs (*n* = 102 per condition) was measured by ImageJ using the scale bar as a reference. In the plots, the horizontal black line indicates the median, while each dot indicates each single value. Data are displayed as mean ± standard deviation from four independent samples. A two-tailed t-test was performed for statistical analysis. Statistical significance is given as: ****(*p* < 0.001). (C) AgNO_3_–stained polyacrylamide gel (12%) of the OMVs isolated from cells grown in ±IPTG. (D) Volcano plot depicting the fold change (FC) and the statistical significance (*p*-value) of the proteome of the OMVs collected from cells grown in ±IPTG. The FC corresponds to −IPTG/+IPTG ratio. Blue and red dots indicate those proteins found significantly decreased or increased (FC ≥ ∣1.5∣; *p* < 0.01), respectively. Black dots represent those proteins either not significantly affected (FC < ∣1.5∣) or with a *p*-value ≥0.01. The projection of some proteins described in the main text is shown. (E) Left: pie chart showing the number of IM proteins, cytoplasmic proteins and proteins with a signal peptide (SPI/SPII) and their relative abundance (%) over the total number of significantly affected proteins. Right: same as left but depicting the number and the relative abundance of the different classes of SPI/SPII proteins over the total number of significantly affected SPI/SPII proteins. (F) Bar chart showing the number of different classes of SPI/SPII proteins, including cytoplasmic proteins, found significantly increased (red) or decreased (blue) in the OMVs upon TamL depletion.

As just described, TamL depletion is accompanied by the shedding of large OMVs (Figure 4A and B). To better understand TamL role in membrane homeostasis, we investigated whether and how OMVs protein composition was affected by TamL depletion by purifying OMVs from cells grown in permissive and non-permissive conditions. We noticed that, at equal cell density (OD_600_) and growth volume, the dry weight of the OMVs isolated from TamL-depleted cells was ten times higher than that of the OMVs from nondepleted cells (60 vs 4–6 mg/L), and these OMVs exhibited an approximately twofold increase in diameter (median: 185 vs 87 nm, Figure 6A and B). The qualitative analysis of the OMVs proteome by AgNO_3_ staining revealed a noticeable difference in the protein band pattern between the OMVs isolated from the two conditions of growth (Figure 6C), which clearly indicates that TamL depletion has a strong impact on the OMVs protein composition. In fact, the MS-based analysis of the OMVs proteome revealed that the abundance of 355 of the identified proteins was significantly affected (FC ≥ ∣1.5∣; *p* < 0.01). 217 (61%) of these proteins were SPI/SPII proteins (Figure 6D and E), while 101 (29%) and 37 (10%) were cytoplasmic and IM proteins, respectively (Tables S5 and S6). Alike in the OM fractions, the SPI/SPII proteins were the most abundant category (Figure S6 and Table S7).

A deeper inspection of the SPI/SPII proteins (Table S5) showed that the predicted periplasmic proteins represented the most numerous class (108 out of 355), followed by OM lipoproteins (54 out of 355), integral/β-barrel OMPs (28 out of 355), and proteins with a T9SS C-terminal sorting domain (26 out of 355). Of the 54 OM lipoproteins, 39 and 14 were predicted to be surface-exposed and periplasmic OM lipoproteins, respectively. A precise localization could not be assigned for two proteins (Figure 6E).

Interestingly, the OMVs isolated in non-permissive conditions were almost exclusively enriched with cytoplasmic and periplasmic proteins. In particular, we observed that the vast majority (100 out of 108) of the periplasmic proteins showed a significant increment upon TamL depletion (Figure 6F). They also stood out as the category of SPI/SPII proteins with the greatest fold change (FC), of which approximately half (48 out of 100) exhibited a FC ≥ 10. Furthermore, the LolA homolog (Fjoh_2111)^49,50^ was found increased (FC: 3.9, Figure 6D), alike what was observed in the OM proteomics analysis.

In contrast, the OMVs released upon TamL depletion were nearly completely devoid of surface-exposed lipoproteins, integral/β-barrel OMPs, and proteins with a T9SS C-terminal sorting domain (Figure 6F). Of these, surface-exposed lipoproteins were the most negatively affected, with 37 out of 39 showing decreased abundance (Figure 6F). Interestingly, while their exact function is unknown, most of these lipoproteins were predicted to have domains involved in carbohydrate/lipid/small molecule-protein binding, likely interacting with integral/β-barrel OMPs involved in the transport and metabolism of these ligands.

As for the integral/β-barrel OMPs, we identified the FadL homolog (Fjoh_4941), which showed decreased abundance (FC: 0.4; Figure 6D).

Proteins with a T9SS C-terminal sorting domain were also negatively affected by TamL depletion (25 out of 26 proteins strongly decreased) (Figure 6F). Most of them belong to Polysaccharide Utilization Loci (PULs)^51^ and have carbohydrate-binding modules (CBMs) and glycosyl hydrolase (GH) domains, which suggests their involvement in carbohydrate transport and metabolism.

Taken together, these results revealed that, under physiological conditions of growth, *F. johnsoniae* produces and releases OMVs that are enriched of integral/β-barrel OMPs, OM lipoproteins, and of proteins predicted to pass through the T9SS, primarily involved in the transport and metabolism of complex sugars and metabolites, or in the homeostasis of cell-wall. In contrast, the loss of OM integrity due to TamL depletion is associated with the abnormal release of large OMVs, whose protein cargo is primarily composed of periplasmic and cytoplasmic proteins.

### TamL unlikely functions as a lipid transporter/insertase in F. johnsoniae

**Figure 7.**
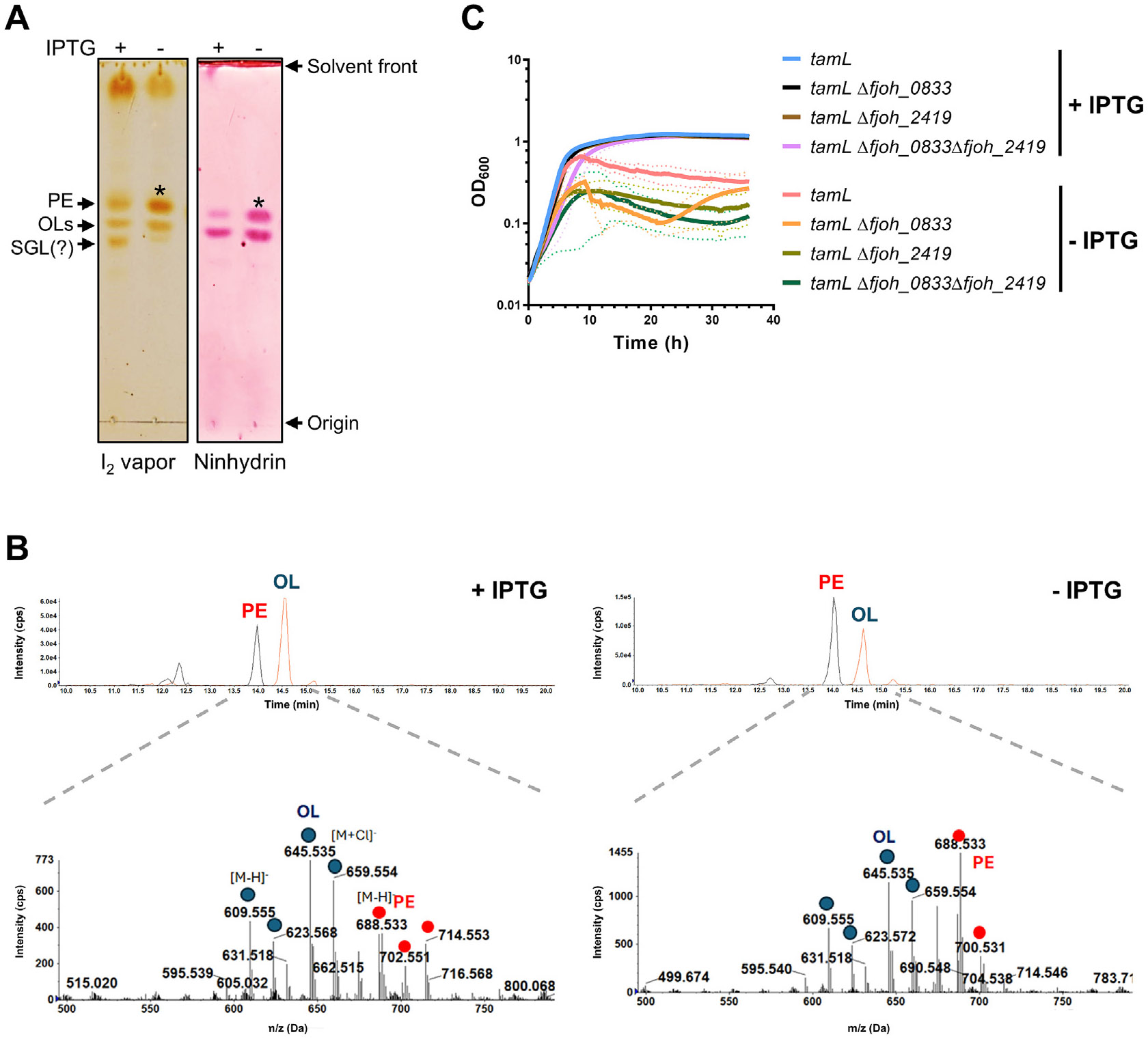
Depletion of TamL affects the lipid content of OMVs and the growth of *F. johnsoniae* mutants deficient in sulfonolipid and ornithine lipid formation. Lipids from OMVs were isolated from cells grown in ±IPTG and analyzed by TLC (A) or LC-MS (B). (A) Lipids were resolved in a solvent mixture of chloroform/methanol/ammonium hydroxide (140:60:10, v/v/v) before being revealed by iodine (I_2_) vapor and ninhydrin staining. A significative phosphatidylethanolamine (PE) increment in the OMVs from −IPTG is observed (labelled “*”). PE and ornithine lipids (OLs) were assigned to the respective spots based on standards. (B) Upper panels: ion chromatograms of the LC-MS analysis of total lipids extracted showing the elution profiles of PE and OLs. Bottom panels: negative ion mode mass spectra of the fractions of the upper panels with retention times between 13.5 and 15 min where PE and OLs were expected to be eluted off the column. Both lipid species were subjected to MS/MS analysis, and the fragmentation confirmed their identity. (C) Growth curves of the *F. johnsoniae* TamL depletion strain, P_ompA_::*lacI*-P_cfxA-lacO_::*tamL* (*tamL*), and of the ornithine lipids and sulfonolipids mutants (*Δfjoh_0833* and *Δfjoh_2419*, respectively) in the TamL-depletion parental strain grown in ±IPTG. Data are shown as mean ± standard deviations from at least three biological replicates.

Lipidomic studies have shown that *F. johnsoniae* OM is mainly composed of sulfonolipids (SLs), ornithine lipids (OLs) and the phospholipid phosphatidylethanolamine (PE), while other minor lipids can be detected depending on the growth conditions.^35,36^ SLs and OLs represent the two dominant lipid classes,^35^ and studies performed on a mutant lacking *fjoh_2419*, encoding an 8-amino-7-oxononanoate synthase needed for SLs biosynthesis, suggest that the balance between SLs and OLs is crucial for the maintenance of the OM permeability.^36^ In the *E. coli* mutant lacking YhdP and TamB, the decrease in the phospholipid anterograde (IM to OM) transport causes an accumulation of LPS in the OM, which then leads to increased OMVs production and release to maintain the proper LPS/phospholipid balance.^25,26^ As our data show that TamL depletion causes blebbing and severely compromises the OM integrity, we wondered if this phenotype was a consequence of lipid asymmetry in the OM. Hence, we first extracted the lipid content of the membrane fractions and OMVs isolated from cells grown in permissive and non-permissive conditions, and we then analyzed their lipid profile by thin layer chromatography (TLC). While in the membrane fractions isolated in the two conditions (±IPTG) we did not observe main changes in the OM lipid pattern (Figure S7), we instead detected more PE in the OMVs from TamL-depleted cells, whereas no substantial changes in the level of OLs could be observed (Figure 7A). The SLs content could not be assessed. Moreover, the iodine vapor staining revealed the presence of a third spot just below that of OLs, which could correspond to serineglycine lipids (SGLs) based on previous results^36^ and the absence of detection by ninhydrin staining (Figure 7A). This spot was barely detectable in the OMVs from TamL-depleted cells, thus indicating that TamL depletion may have a potential impact also upon these lipid species.

We then analyzed the OMVs lipid profile by LC-MS to gain more insights. Although the MS analysis did not provide an absolute quantification, we found that the PE/OLs ion intensities were higher in the OMVs from TamL-depleted cells, in support of the TLC results (Figure 7B). Additionally, the analysis detected elevated levels of SLs in both sample types (i.e., OMVs from cells grown with ±IPTG), though no difference was observed. Therefore, we could conclude that TamL depletion affects the PE/OLs balance in the OMVs, likely determining an increase in the PE levels, whereas no major effects are seen on OLs and SLs. Next, we checked the levels of LPS in both the OM fractions and the OMVs and we found that TamL depletion does not seem to cause a significative alteration of the LPS amount in neither the OMVs nor the OM fractions (Figures S8A and B).

If TamL is required for the transport/insertion of SLs and/or OLs in the OM, mutants unable to synthesize either or both lipids would result in the loss of cell viability to a same extent as that observed upon TamL depletion. To prove this, we generated single and double mutants lacking *fjoh_2419* and *fjoh_0833* (encoding a bifunctional acyltransferase responsible for OLs).^36,52^ Interestingly, in CYE medium no substantial differences could be observed compared to the WT strain, which indicates that the co-deletion of *fjoh_0833* and *fjoh_2419* has no effect on cell fitness and does not replicate the loss of cell viability observed upon TamL depletion (Figure S9). In contrast, in MM, only the double mutant *Δfjoh_0833Δfjoh_2419* displayed a significative growth reduction (Figure S9), which may be attributed to the fact that in this strain no other major lipid classes can fully complement the lack of both OLs and SLs.

We then performed the same experiment using the same mutants for *fjoh_0833* and *fjoh_2419* but in the TamL depletion strain. If TamL is the OLs/SLs transporter, *fjoh_0833* and/or *fjoh_2419* are in epistatic interaction with *tamL*, that is, their deletion should not worsen the reduced growth fitness observed upon TamL depletion. In contrast, we observed that both single mutants for *fjoh_0833* and *fjoh_2419* exhibited a drastic growth reduction upon TamL depletion compared to the control strain, and this phenotype was exacerbated in the double mutant (*Δfjoh_0833Δfjoh_2419*) (Figure 7C).

Furthermore, except for OLs in the *Δfjoh_0833* and *Δfjoh_0833Δfjoh_2419* mutants, the whole-cell lipid profiles of the *Δfjoh_0833* and *Δfjoh_2419* mutants in the TamL depletion background did not show substantial differences compared to the reference strain (*tamL*) in neither permissive or non-permissive conditions of growth (Figure S10).

Altogether, the above findings do not support the hypothesis that TamL may serve as OLs/SLs transporter/insertase. Therefore, we can conclude that TamL is most likely not implicated in the transport of the main OM lipid classes in *F. johnsoniae*.

### Physical interaction between TamL and TamB

So far, our data do not seem to support the hypothesis that TamL is implicated in the anterograde transport of OM lipids. We then wondered if TamL truly establishes a physical interaction with TamB *in vivo*. Therefore, we performed pull-down experiments after crosslinking with DTSSP using (3xFLAG)TamL as a bait. MS data analyses confirmed the presence of TamB as the most abundant protein after TamL only in the (3xFLAG)TamL-elutions (Table S8). The pull-down assays did not allow us to identify with statistical significance OMPs that co-purified exclusively with TamL. At the current state, we cannot rule out the possibility that TamL, upon interaction with TamB, may transport OMPs.

We then sought to simulate TamL-TamB putative interaction using AlphaFold Multimer.^53^ Interestingly, the C-terminal pseudosubstrate domain of TamB (residues 1321–1420) was predicted to interact via β-strand augmentation with the last β-strand of the β-barrel domain of TamL in a similar manner to that proposed between TamA and TamB,^19^ and also between BamA and its OMP substrates.^54-56^ Furthermore, a similar interaction has been recently proposed to occur between the C-terminus of YdbH, an AsmA-like protein, and the OM lipoprotein YnbE.^29^ Based on the *in silico* prediction, the interaction between TamL and TamB would create a 22 stranded hybrid β-barrel internally crossed by a small fragment of TamB C-terminus (Figure S11A).

### TamL, TamB and TamA are conserved in Bacteroidota

**Figure 8.**
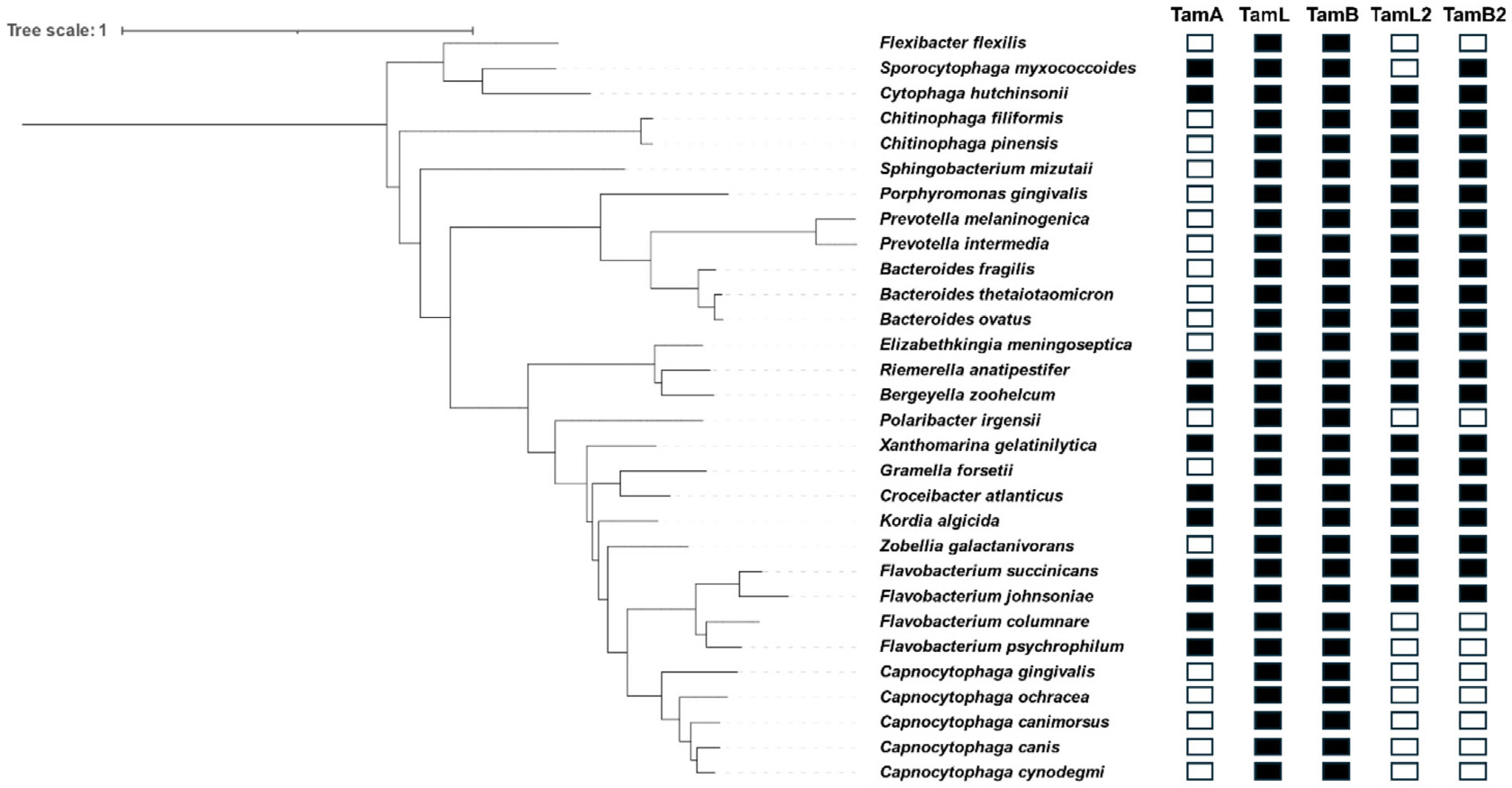
Conservation of TAM proteins in Bacteroidota. The sequences of TamA, TamL, TamB, TamL2 and TamB2 from *F. johnsoniae* were used to identify their respective homologs in Bacteroidota by DELTA-BLAST.^42^ A phylogenetic tree was then generated via phyloT^87^ based on NCBI taxonomy. Black and white squares indicate that the presence or absence of the protein homolog, respectively.

The finding that *F. johnsoniae* possesses multiple TamL and TamB, as well as TamA, led us to investigate whether this genetic feature could be shared by Bacteroidota in general. To this aim, we performed an *in silico* analysis searching for TamA, TamL, TamL2, TamB and TamB2 homologs in representative genera and species of the Bacteroidota phylum using *F. johnsoniae* protein sequences as queries (Figure 8 and Table S9). Strikingly, we found that all the species investigated possess TamL and TamB homologs. In 20 out of 30 of these species, we also identified TamB2 and TamL2 proteins, encoded in a operon as in *F. johnsoniae* (Figure 8).

Concerning TamA, we identified homologs in eleven species (Figure 8 and Table S9). We consider this finding remarkable as it reveals the presence of TamA in other Bacteroidota members in addition to *F. johnsoniae*, thus confirming that TamA is not only confined to Proteobacteria as previously thought.^30^

In summary, our *in silico* analysis reveals that, while TamL and TamB are conserved in all Bacteroidota we analyzed, TamL2 and TamB2 as well as TamA are restricted to some species.

### TamL essentiality and its interaction with TamB is not solely restricted to F. johnsoniae

**Figure 9.**
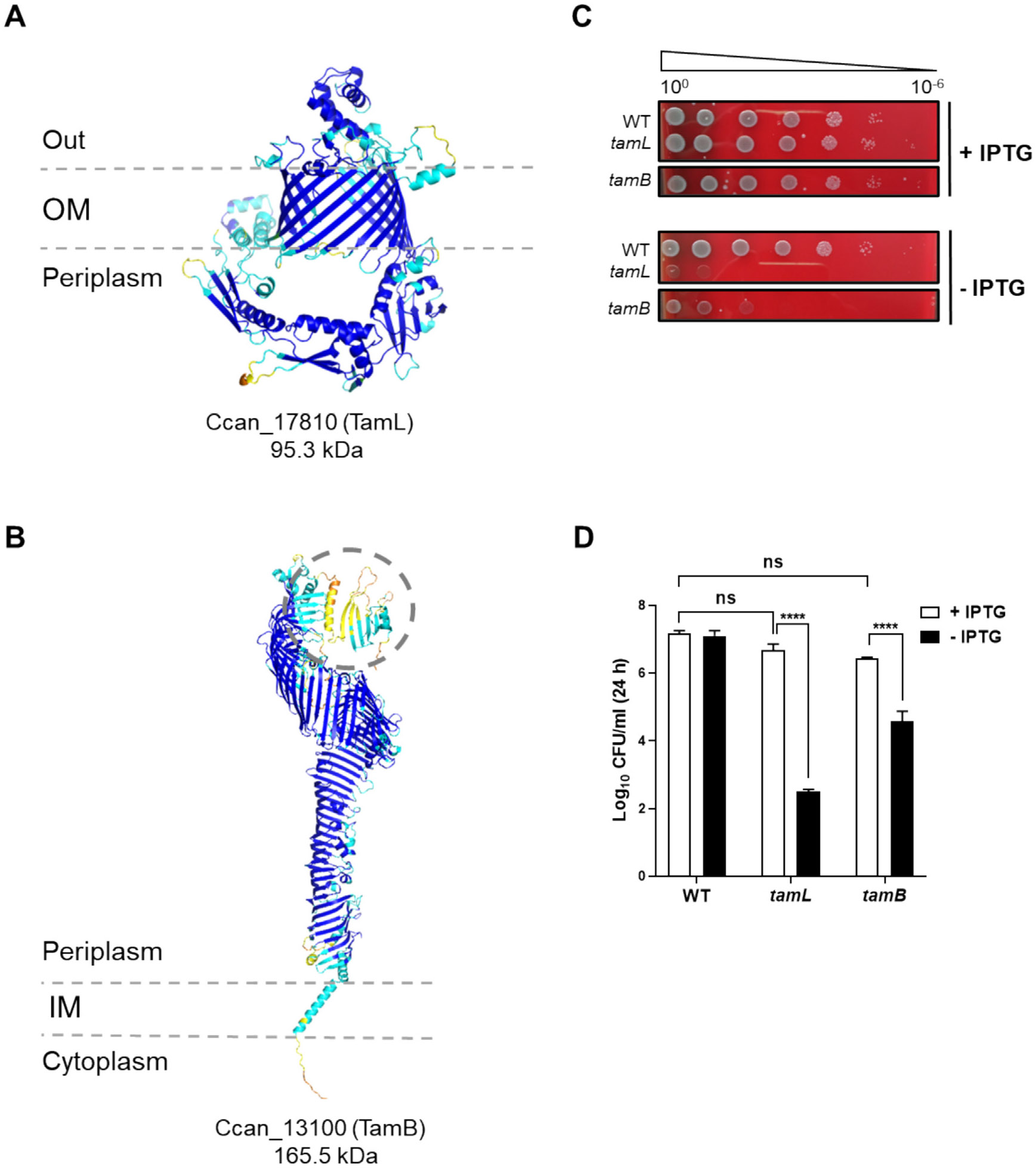
*C. canimorsus* possesses one TamL and one TamB. (A and B), three-dimensional structures of TamL (A) and TamB (B) homologs in *C. canimorsus* as predicted by AlphaFold2.^86^ Protein names and predicted molecular weights are reported below each structure. Structures are colored based on the per-residue confidence score (pLDDT) between 0 and 100: dark blue (pLDDT > 90), cyan (90 > pLDDT > 70), yellow (70 > pLDDT > 50), and orange (pLDDT < 50). In (A), the N-terminal lipid moiety that likely anchors TamL to the OM is not shown. In (B), the predicted C-terminal pseudosubstrate domain is highlighted via a dashed circle. (C and D), effect of TamL depletion on cell viability in *C. canimorsus*. (C) Spot assay of wild-type (WT), P_ompA_::*lacI*-P_cfxA-lacO_::*tamL* (*tamL*) and P_ompA_::*lacI*-P_cfxA-lacO_::*tamB* (*tamB*) strains grown in SB (±IPTG). Plates were incubated at 37 °C, 5% CO_2_ and photographed after 48 h. (D) End-point growth of the same strains as in (C) in heat-inactivated human serum. After 24 h of growth, cells were plated on SB to enumerate the CFUs/ml. Data were then Log_10_-transformed and displayed as mean ± standard deviation from at least three independent experiments. A one-way ANOVA followed by Tukey’s multiple comparison test was performed. Statistical significance is displayed as following: ns = not significant; ****p < 0.0001.

As pointed out by our *in silico* analysis, *C. canimorsus*, a commensal bacterium of cats and dogs mouth and a human pathogen, whose genus is closely related to that of *Flavobacterium*,^41^ possesses only one TamL homolog (Protein accession: WP_042002088.1; Table S9) and one TamB homolog (Protein accession: WP_095900346.1; Table S9) encoded by the genes *Ccan_17810* and *Ccan_13100*, respectively. Notably, TamB is the only AsmA-like protein that we could identify in *C. canimorsus*. Through the inspection of their AlphaFold2-based predicted structures, we could confirm their identity as TamL and TamB homologs (Figure 9A and B), where Cys20 of TamL is the predicted site of lipidation (likelihood > 0.99). Unlike TamB of *F. johnsoniae* (Figure 1B), the C-terminal end of the TamB homolog of *C. canimorsus* is not portrayed as disordered (Figure 9B).

Similarly to *F. johnsoniae*, attempts to delete *tamL* and *tamB* of *C. canimorsus* were unsuccessful, supporting their essential role.

We then generated TamL and TamB depletion strains using the same strategy as for *F. johnsoniae*. As shown in Figures 9C and 9D, we could observe a strong reduction in cell viability upon TamL or TamB depletion under non-permissive conditions compared to permissive ones on plate and liquid medium (SB plates and heat-inactivated human serum, respectively). Interestingly, TamL depletion turned out to be more deleterious than TamB one. We therefore conclude that TamL and TamB are also essential in *C. canimorsus*.

Finally, similarly to what was observed in *F. johnsoniae*, the pseudosubstrate domain of TamB (residues 1346–1149) was predicted in the AlphaFold Multimer model to establish physical interactions with the last strands of TamL β-barrel (Figure S11B), thus indicating that such a mechanism of interaction could be typical of the TAM complexes from different bacteria.

## Discussion

Through this work we aimed to provide new insights into the molecular function of TamL in the environmental Bacteroidota *F. johnsoniae*.^37,39,46,48^

Our data revealed the existence of multiple TamL (Fjoh_1464 and Fjoh_1900) and TamB (Fjoh_4592 and Fjoh_1899) homologs, as previously reported in the Bacteroidota *Porphyromonas gingivalis*.^57^ Moreover, we also identified a TamA homolog (Fjoh_0402), which to our knowledge has never been identified outside the Proteobacteria phylum. We found that Fjoh_1464 and Fjoh_4592 are essential, and that the lipidation occurring at the N-terminal end of Fjoh_1464 during its maturation is crucial for cell viability. Whether the lipidation is needed for Fjoh_1464 OM localization or its activity remains unknown. This finding was unexpected as, to our knowledge, there are no previous reports on the essentiality of the TAM subunits, with the only exception of the TamB homolog in *B. burgdorferi*.^31^ We named Fjoh_1464 and Fjoh_4592 TamL and TamB, respectively; TamL2 and TamB2 were instead assigned to the non-essential Fjoh_1900 and Fjoh_1899, respectively. Currently, we do not have insights into what the function of TamL2 and TamB2 within the cell could be. This will be the object of future investigations.

We observed that TamL and TamB do not show the same pattern of temporal regulation since TamL presence within the cell, unlike TamB, seems to be restricted to the exponential phase of growth. A possible explanation is that an active interaction between the two proteins, as evidenced by our pull-down assays (see below), might be specifically required in actively growing cells. Consequently, the structural complexity of TamB, which spans the periplasm and the peptidoglycan (PG) sacculus, might make it more advantageous to downregulate TamL in order to deactivate the TAM.

We then generated a TamL depletion strain, but we failed to obtain a TamB depletion one. At present, we cannot explain why, though the genomic region where the *tamB* gene is located may play a role. Using the TamL depletion strain, we confirmed its essentiality in liquid medium and on plate, which was accompanied by morphological defects, cell aggregation and increased budding of large OMVs. Furthermore, TamL depleted cells exhibit increased sensitivity to several OM perturbing agents, such as Polymyxin B, Vancomycin and SDS. Notably, cell fitness in TamL-depleted cells could be ameliorated by increasing the concentration of Mg^2+^ to 30 mM. Similar phenotypes were previously observed in *E. coli* and *Brucella suis*.^25,26,44,45,58^ It is important to highlight that these results were restricted to TamB mutants, whereas our study is the first to apply them specifically to TamL.

We also found that TamL depletion significantly altered the OM proteome determining a sharp increase in OM lipoproteins. We indeed observed a strong enrichment in the LolA (Fjoh_2111, FC: 3.0; Fjoh_1085, FC: 5.7) and LolB (Fjoh_1084, FC: INF) homologs, which are members of the lipoprotein trafficking system, recently investigated in *F. johnsoniae*.^49^ Although the observed increase in OM lipoproteins upon TamL depletion is intriguing, the function of most of them remains unclear. It must be recalled here that OM lipoproteins are of crucial importance in bacterial survival, especially in maintaining the integrity and functionality of the OM under stress conditions. Noticeable examples are the BAM accessory lipoproteins, the Braun lipoprotein (Lpp), and LolB of the Lol system, whose increased abundances were shown to be the result of a stress response to OM damage.^59-61^ Therefore, it is plausible that TamL depletion disrupts OM integrity, thus triggering a stress response that leads to an increase in OM lipoproteins. In fact, it should be recalled that TamL is a lipoprotein itself and our data clearly support its involvement in OM stability.

On the other hand, we did not observe an overall downregulation of integral/β-barrel OM proteins, as one might expect from the depletion of a foldase/insertase involved in their transport and folding. Prior studies have instead shown that the depletion of BamA and BamD, the two essential subunits of the BAM complex (the main OMP assembly factor in diderm bacteria), were accompanied by a significant reduction in the OM protein content.^6,62-64^ Therefore, our proteomics data would not support the hypothesis that TamL functions as a general OM foldase/insertase in *F. johnsoniae*.

We found that the OMVs released from TamL-depleted cells were twice as big as the control strain, highly enriched in periplasmic proteins, and almost completely devoid of OMPs. This finding reinforces our model of TamL as a crucial player in maintaining OM integrity since its depletion destabilizes the OM and leads to the release of no physiological OMVs lacking their functional protein cargo. Strikingly, we observed that surface-exposed lipoproteins, found enriched in the OM fractions, were also reduced in the OMVs. Since these lipoproteins constitute a significant portion of OMVs cargo in Bacteroidota,^65,66^ we cannot rule out that TamL may be required directly/indirectly to their incorporation into OMVs.

Considering all the above results, possible contamination coming from OMVs in the OM fractions cannot be completely excluded. However, we believe that such contamination is negligible and unlikely to significantly impact the proteomics analyses due to the mechanical and chemical steps employed to isolate the OM fractions.

Except for a slight increment in phosphatidylethanolamine (PE) in the OMVs, we did not observe any significant impact on the main OM lipid content in TamL-depleted cells. Furthermore, in the TamL depletion strain the deletion of *fjoh_2419* and *fjoh_0833*, responsible for the biosynthesis of sulfonolipids (SLs) and ornithine lipids (OLs), respectively,^36,52^ did not result in the same loss of cell viability observed in the parental strain. Instead, the deletion of *fjoh_2419* and *fjoh_0833* further impaired cell fitness, suggesting an additive effect on the OM instability. Based on these findings, we conclude that TamL is most likely not involved in OM lipid transport in *F. johnsoniae*.

We confirmed that TamL and TamB establish physical interaction *in vivo*. Assuming that TamL and TamB form the TAM complex in *F. johnsoniae*, this TAM may operate similarly to its counterpart in Proteobacteria, with TAM substrate(s) passing through TamB before reaching TamL.^13-15^ Consequently, the loss of TamL could result in the stalling of TAM substrate (s) on TamB, which could ultimately be detrimental for cell viability by depriving cells of a functional TamB. In this context, the upregulation of TamB we observed upon TamL depletion may represent a cellular attempt to cope with the stalling of substrate(s) on TamB.

Our *in silico* analysis indicates that TamL and TamB are widely conserved in Bacteroidota while TamL2, TamB2 and TamA are restricted to some species. We could show that in another Bacteroidota, *C. canimorsus*, a human pathogen primarily recognized as a commensal in the oral cavities of cats and dogs,^41^ TamL and TamB homologs are essential and the AlphaFold2-based model predicts their interaction. Moreover, the finding that in *C. canimorsus* TamL depletion is more deleterious than TamB is intriguing, and further work is needed to understand the reason.

Altogether, these findings suggest that both TAM subunits may be also essential in other Bacteroidota species. Likewise, the identification of the TamA homologs in other Bacteroidota clearly suggests that TamA and TamL co-existence in the OM is not restricted to *F. johnsoniae*.

The molecular contribution of TamL to OM integrity remains to be fully understood. Several possibilities may be proposed. For example, TamL may be required to fold and insert in the OM one or more proteins that are essential for the stability of the OM. Additionally, the POTRA domains of TamA in *P. aeruginosa* have been recently shown to establish interaction with the polar heads of phospholipids, thus modulating membrane properties.^17^ We do not exclude that the same interaction might occur between TamL and the OM lipids.

## Materials and Methods

### Bacterial strains and media

*Flavobacterium johnsoniae* UW101 WT (ATCC 17061) and derivative strains were grown in Casitone-Yeast Extract (CYE) medium or motility medium (MM) at 30 °C.^67,68^ *Capnocytophaga canimorsus* 5 strains were grown on either heart infusion agar (Difco) supplemented with 5% defibrinated sheep blood (ThermoFisher) plates (SB plates) or in heat-inactivated human serum (HIHS) at 37 °C in the presence of 5% CO_2_.^33^ *Escherichia coli* strains were grown in Lysogeny Broth (LB) medium at 37 °C. All strains used in this study are listed in Table S10. When needed, the following antibiotics were used: ampicillin (100 μg/ml); streptomycin (100 μg/ml), gentamicin (20 μg/ml), erythromycin (100 μg/ml); tetracycline (20 μg/ml).

### Strain construction

Plasmids and oligonucleotides used in this study are listed in Tables S11 and S12, respectively. Plasmids for mutagenesis of *F. johnsoniae* were generated by either Gibson assembly,^69^ or by sequential cloning into the non-replicative plasmid pYT354 (Table S11). In the former case, plasmid pYT354 and around 2000 base pairs (2 kb) upstream and downstream the region of interest were PCR-amplified. All the PCR-generated fragments were then assembled by Gibson assembly and cloned in *E. coli* Top10 by electroporation.

All the generated suicide plasmids were introduced in the appropriate *F. johnsoniae* background strain by triparental mating, using *E. coli* Top10 and *E. coli* MT607 as donor and helper strains, respectively (Table S10). Erythromycin resistance was used to select colonies with chromosomally integrated plasmid. After PCR screening, one colony was picked and incubated in 5 ml LB overnight at 30 °C to promote plasmid loss, and then plated onto LB agar plates containing 5% (w/v) *D*-sucrose. Plates were incubated for 2–3 days at 30 °C until single colonies were visible. Colonies resistant to *D*-sucrose were selected and PCR-screened for the presence of the desired genomic mutation. When needed, 1 mM isopropyl-beta-D-thiogalacto pyranoside (IPTG; ThermoFisher Scientific) was added to the plates.

The same approach was used to generate *C. canimorsus* strains, though with some differences: (i) only 600 base pairs upstream and downstream the region of interest were PCR-amplified; (ii) after PCR screening, one colony was picked and streaked onto a SB plate; (iii) SB plates containing 3% (w/v) *D*-sucrose were used.

### Construction of a TamL depletion strain

We generated the P_ompA_::*lacI* construct (Table S11) by putting the gene coding for the LacI transcription repressor (*lacI*) under the regulation of the promoter of *fjoh_0697*, encoding the outer membrane protein OmpA. This construct (P_ompa_::*lacI*) was then cloned in pYT354 carrying the homologous regions to *fjoh_0061* and *fjoh_0062*, thus generating the plasmid pYT354-P_ompA_::*lacI* (Table S11) which allowed the insertion of this construct in the genomic locus between *fjoh_0061* and *fjoh_0062*. Then, we engineered the constitutive promoter of the cefoxitin resistance gene (P_cfxA_)^70^ by adding the binding sequences of the LacI transcription repressor (*lacO_3_* and *lacO_1_*) upstream of the −33 binding box and downstream of the transcriptional initiation site (TIS) of P_cfxA_. Furthermore, upstream of lacO_3_ we added the transcription terminator site of *fjoh_0014* (*ter_fjoh_0014_*), thus generating the *ter_fjoh_0014_-lacO_3_*-P_cfxA_-*lacO_1_* construct (P_cfxA-lacO_). Then, we cloned *tamL* or *tamB* downstream of this construct to generate a strain where their native promoters were replaced with P_cfxA-lacO_ (Table S10).

The same approach was used to generate TamL and TamB depletion strains in *C. canimorsus* (Table S10) with the only difference that the P_ompa_::*lacI* construct was inserted in the genomic locus between *Ccan_09290* and *Ccan_09300*.

### Assessment of TamL and TamB profile during cell growth

*F. johnsoniae* double-tagged strain ((3xFLAG) *tamL*/(2xStrep)*tamB*) was inoculated at OD_600_:0.05 in CYE (10 ml) and incubated at 30 °C, 160 rpm for 24 h (pre-culture). Then, the OD_600_ was measured, and accordingly bacterial cells were inoculated in CYE (25 ml) at OD_600_:0.05, and incubated at 30 °C, 160 rpm. At defined time points, samples were collected for immunoblot and phase-contrast microscopy analyses.

The same protocol was applied to grow the *F. johnsoniae* TamL depletion strain (P_ompa_::*lacI*-P_cfxA-lacO_-*tamL*), though IPTG (1 mM) was added to the pre-culture medium (permissive condition). Afterwards, the pre-culture was inoculated in two flasks: one with 1 mM IPTG (permissive condition) and one without (non-permissive condition).

### Determination of bacterial growth upon TamL depletion and outer membrane permeability assays

*F. johnsoniae* TamL depletion strain (P_ompA_::*lacI*-P_cfxA-lacO_-*tamL*) was grown overnight in CYE (1 mM IPTG) at 30 °C at 160 rpm. A culture volume corresponding to OD_600_:1 was harvested at 5,000g for 5 min at RT. Bacterial pellet was washed in 1 ml of 1x PBS, and then harvested again. Cells were diluted to OD_600_: 0.05 in 1 ml of fresh CYE or MM, ±IPTG (1 mM). 200 μl of cells at OD_600_: 0.05 was deposited onto a 96-well microplate and growth was monitored for 36 h with continuous shaking at 30 °C, in an automated plate reader (Bioscreen C, Lab Systems) measuring the OD_600_ every 10 min.

For spot assay, cells were OD_600_-adjusted and washed as above before being serial diluted in 1x PBS. 3 μl from serial dilutions were deposited onto CYE-Agar plates (±IPTG). Plates were incubated at 30 °C and photographed after 48 h. For stress tolerance assays, cells were resuspended in fresh CYE medium in the presence of the following (freshly made) compounds: Polymyxin B (40 μg/ml), SDS (0.0025%, w/v), and Vancomycin (62.5 μg/ml). Data are displayed as mean ± standard deviation (SD) of at least three biological replicates.

For *C. canimorsus*, cells grown on SB plates (with 1 mM IPTG for the TamL/TamB depletion strains) were collected and resuspended in 1x PBS for OD_600_ measurement. After normalizing to OD_600_:1, bacteria were serially diluted in 1x PBS until 10^−6^. 3 μl from serial dilutions were spotted on SB plates. Plates were incubated at 37 °C and 5% CO_2_. Pictures were taken after 24 h.

### End point growth of C. canimorsus in heat-inactivated human serum (HIHS)

*C. canimorsus* cells grown on SB plates (with 1 mM IPTG for the TamL/TamB depletion strains) were collected and resuspended in 1x PBS. After normalizing to OD_600_: 0.2, bacteria were serially diluted in 1x PBS until 10^−4^. 20 μl of this dilution was added to a 96-wells plate already filled with 180 μl of HIHS (containing 1 mM of IPTG when needed) so that each well contained around 2 × 10^2^ cells. Three technical replicates per condition were performed. The 96-wells plate was incubated without agitation at 37 °C and 5% CO_2_ for 24 h. To determine the colony forming units (CFUs), serial dilutions were plated on SB plates (containing 1 mM IPTG when needed) after 24 h. SB plates were incubated 3–4 days before CFUs counting.

### Separation of the inner membrane and outer membrane

Alike what described in *F. psychrophilum*,^71^ previous attempts to separate membrane fractions by sucrose gradient ultracentrifugation were not fruitful. We therefore adapted a previously described protocol where the ionic detergent Sarkosyl is used to selectively solubilize the inner membrane while conserving the integrity of the outer membrane.^72^ The workflow after cell lysis is depicted in Figure S3. Briefly, cells were grown in CYE (10 ml) containing IPTG (0.25 mM) at 30 °C for 12 h at 160 rpm (pre-culture). A culture volume corresponding to OD_600_:2 was harvested at 5,000*g* for 5 min at RT, washed in 1x PBS, and harvested as before. Afterwards, cells were inoculated at OD_600_:0.05 in two flasks containing CYE (50 ml) at 30 °C, 160 rpm. IPTG (1 mM) was added to just one of the two flasks for the permissive condition of growth. After 12 h of growth, the volume equivalent of OD_600_:40 was harvested at 7,000*g* for 10 min at 4 °C. Bacterial pellet was resuspended on ice in a solution consisting of 10 mM HEPES, pH 7.4, 1 mM EDTA (containing 1 tablet of EDTA-free protease inhibitors). Cells were lysed through a cell disrupter (Constant Systems) at 35,000 psi. Cell debris and unlysed cells were removed by centrifugation at 2,500*g* for 10 min at 4 °C. Then, cell extracts were centrifuged at 108,000*g* for 107 min at 4 °C using an ultracentrifuge device equipped with a TLA 100.3 fixed-angle rotor (Beckman). The supernatants were retained as soluble fractions while the pellets, corresponding to the total membrane fractions (i.e., inner and outer membranes together), were resuspended in 2 ml of a fresh solution consisting of 10 mM HEPES, pH 7.0. The total membrane fractions were further fractionated by partial solubilization in 2% (w/v) sodium n-lauroylsarcosinate solution on a rotating wheel for 1 h at RT, followed by a second round of ultracentrifugation performed as above. Pellets (outer membranes) were resuspended in 250 μl of ice-cold 10 mM HEPES, pH 7.4. All samples were temporarily stored at 4 °C.

### Assessment of membrane sample purity

The succinate dehydrogenase (SDH) activity was measured as described elsewhere.^73^ Briefly, the protein concentration of the different membrane fractions was quantified using the Quick StartTM Bradford Protein Assay (Bio-rad). Accordingly, a 96-well plate was filled with 100 μl of sample, corresponding to 24 μg of total proteins, or dH_2_O. 60 μl of the following freshly made reaction mix was then added to each well: 50 mM Tris-HCl, pH 8.0, 4 mM KCN and 40 mM disodium succinate. After 5 min of incubation at RT, 20 μl of 4 mM DCIP and 20 μl of 2 mM PMS were subsequently added. For the detection of the SDH activity, the absorbance at 600 nm was measured using a SpectraMax ID3 Molecular Devices fluorimeter every 30 s for 1 h at 25 °C (Figure S12).

To examine the total protein profile of cell fractions, 2 μg of total proteins were loaded onto 12% SDS-PAGE and then visualized by AgNO_3_ staining.^74^

### Proteomic analyses by mass spectrometry

#### Sample preparation.

For the outer membrane proteome, 25 μg of total proteins in 5 mM HEPES pH 7.4 from 5 independent biological replicates were given for protein digestion. For the OMVs proteome, 20 μg of total proteins in 1x Laemmli buffer from 4 independent biological replicates were given. For the proteomic analysis of the elution fractions from pull-down assays, 25 μg of total proteins in 1x PBS from 2 independent biological replicates was given. Prior to protein digestion, the chemical crosslinker (DTSSP) was cleaved by adding 40 mM DTT and incubating at 37 °C for 30 min.

#### Protein digestion.

The samples were treated using Filter-Aided Sample Preparation (FASP) using the following protocol. Briefly, to first wash the filter, 100 μl of 1% formic acid were placed in each Millipore Microcon 30 MRCFOR030 Ultracel PL-30 before centrifugation at 14,500 rpm for 15 min. Proteins were resuspended in 150 μl of urea buffer 8 M (urea 8 M in buffer Tris 0.1 M, pH 8.5), placed individually in a column, and centrifuged at 14,500 rpm for 15 min. The filtrate was discarded, and the columns were washed three times by adding 200 μl of urea buffer followed by a centrifugation at 14,500 rpm for 15 min. For the reduction step, 100 μl of dithiothreitol (DTT) were added and mixed for 1 min at 400 rpm with a thermomixer before an incubation of 15 min at 24 °C. Samples were then centrifugated at 14,500 rpm for 15 min, the filtrate was then discarded, and the filter was washed by adding 100 μl of urea buffer before another centrifugation at 14,500 rpm for 15 min. An alkylation step was performed by adding 100 μl of iodoacetamide (IAA, in urea buffer) in the column and mixing at 400 rpm for 1 min in the dark before an incubation of 20 min in the dark and a centrifugation at 14,500 rpm for 10 min. To remove the excess of IAA, 100 μl of urea buffer were added and the samples were centrifugated at 14,500 rpm for 15 min. To quench the rest of IAA, 100 μl of DTT were placed on the column, mixed for 1 min at 400 rpm and incubated for 15 min at 24 °C before centrifugation at 14,500 rpm for 10 min. To remove the excess of DTT, 100 μl of urea buffer were placed on the column and centrifuged at 14,500 rpm for 15 min. The filtrate was discarded, and the column was washed three times by adding 100 μl of sodium bicarbonate buffer 50 mM (ABC, in ultrapure water) followed by a centrifugation at 14,500 rpm for 10 min. The last 100 μl were kept at the bottom of the column to avoid any evaporation in the column. The digestion process was performed by adding 80 μl of mass spectrometry grade trypsin (1/50 in ABC buffer) in the column and mixed at 400 rpm for 1 min before an incubation overnight at 24 °C in a water saturated environment. The Microcon columns were placed on a LoBind tube of 1.5 ml and centrifuged at 14,500 rpm for 10 min. 40 μl of ABC buffer were placed on the column before centrifugation at 14,500 rpm for 10 min. Trifluoroacetic acid (TFA) 10% in ultrapure water were added to the contain of the LoBind Tube to obtain 0.2% TFA. The samples were dried in a SpeedVac up to 20 μl and transferred to an injection vial.

#### Mass Spectrometry (MS).

The digest was analyzed using nano-LC-ESI-MS/MS tims TOF Pro (Bruker, Billerica, MA, USA) coupled with an UHPLC nanoElute2 (Bruker). The different samples were analyzed with a gradient of 60 min. Peptides were separated by nanoUHPLC (nanoElute2, Bruker) on a 75 μm ID, 25 cm C18 column with integrated CaptiveSpray insert (Aurora, ionopticks, Melbourne) at a flow rate of 200 nl/min, at 50 °C. LC mobile phases A was water with 0.1% formic acid (v/v) and B ACN with formic acid 0.1% (v/v). Samples were loaded directly on the analytical column at a constant pressure of 800 bar. The digest (1 μl) was injected, and the organic content of the mobile phase was increased linearly from 2% B to 15% in 22 min, from 15% B to 35% in 38 min, from 35% B to 85% in 3 min. Data acquisition on the tims TOF Pro was performed using Hystar 6.1 and timsControl 2.0. tims TOF Pro data were acquired using 160 ms TIMS accumulation time, mobility (1/ K0) range from 0.75 to 1.42 Vs/cm^2^. Mass-spectrometric analyses were carried out using the parallel accumulation serial fragmentation (PASEF)^75^ acquisition method. One MS spectra followed by six PASEF MSMS spectra per total cycle of 1.16 s.

#### Data analysis.

Data analysis was performed using Mascot 2.8.1 (Matrix Science). For database searching, tandem mass spectra were extracted, charge state deconvoluted and deisotoped by Data analysis (Bruker) version 5.3. All MS/MS samples were analyzed using Mascot (Matrix Science, London, UK; version 2.8.1). Mascot was set up to search the *Flavobacterium johnsoniae* UW101 proteome from Uniprot (220718-5021 entries) and a contaminants database, assuming the digestion enzyme trypsin with two missed cleavages. Mascot was searched with a fragment ion mass tolerance of 0.050 Da and a parent ion tolerance of 15 ppm. Carbamidomethyl of cysteine was specified in Mascot as fixed modifications. Oxidation of methionine, deamidation of asparagine and glutamine and acetyl of the n-terminus were specified in Mascot as variable modifications.

For protein identification, Scaffold (version Scaffold_5.1.1, Proteome Software Inc., Portland, OR) was used to validate MS/MS based peptide and protein identifications. Peptide identifications were accepted if they could be established at greater than 96.0% probability to achieve a False Discovery Rate (FDR) less than 1.0% by the Scaffold Local FDR algorithm. Protein identifications were accepted if they could be established at greater than 5.0% probability to achieve an FDR less than 1.0% and contained at least 2 identified peptides. Protein probabilities were assigned by the Protein Prophet algorithm (Nesvizhskii, Al et al Anal. Chem. 2003;75 (17):4646–58). Proteins that contained similar peptides and could not be differentiated based on MS/MS analysis alone were grouped to satisfy the principles of parsimony. Proteins sharing significant peptide evidence were grouped into clusters.

For the analysis of the outer membrane and OMVs proteomes, a t-test (without correction) was performed to compare the means from samples obtained in ±IPTG. When protein peptides could be detected in only one of the two conditions, a representative value of ∣64∣ was used, while for those proteins whose *p*-value was <0.0001, a representative value of 0.00009 was used. The Fold Change (FC) was derived using the permissive condition (+IPTG) as a reference. Proteins exhibiting a FC ≥ ∣1.5∣ and a *p*-value of <0.01 were considered as significant.

### In silico characterization of the proteins identified by MS

Predicted localization and topology analysis for proteins identified by MS were performed using UniProt database, SignalP 6.0,^43^ PSORTb 3.0^76^ and CAZy database.^77^ All predicted lipoproteins were manually checked for the presence of the lipoprotein export signal (LES).^34^ When present, they were assigned as surface-exposed lipoproteins; when absent, they were assigned as periplasm-facing lipoproteins.

### Outer membrane vesicles (OMVs) purification and protein quantification

*F. johnsoniae* cultures were grown as described in the membrane fractionation protocol with the sole difference that the volume of the cultures was increased up to 1 L and 200 ml for cultures in permissive (+IPTG) and non-permissive (−IPTG) conditions, respectively. After 12 h of growth, bacterial cells were pelleted twice at 5,000*g* for 10 min at 4 °C, and then the culture supernatants were passed through a 0.22 μm pore-size filter (Sarstedt) to remove any cell leftover or debris. Afterwards, supernatants were concentrated down to 3 ml using Amicon^®^ Ultra-15 Centrifugal Filter Unit (50 kDa cutoff, Millipore) centrifuging at 3,500*g* for 10 min at 4 °C each time. The concentrated supernatants were then centrifuged at 108,000*g* for 3 h at 4 °C as described above. Pellets (OMVs) were weighed and stored at −80 °C until use.

To quantify the protein content, OMVs were resuspended in 1x Laemmli buffer and boiled at 99 °C for 5 min. Afterwards, the protein concentration was estimated by Pierce BCA protein assay (ThermoFisher Scientific). To get rid of the SDS interference, the ionic detergent compatibility reagent (ThermoFisher Scientific, product reference: 22663) was used in agreement with the manufacturer’s instructions. The OMVs protein profile was examined as for membrane fractions.

### Total lipid extraction and analyses from lipid fractions and OMVs

#### Lipid extraction.

Lipids from membrane fractions (6–8 mg, dry weight) were extracted following a previously described protocol.^78^ For OMV and whole-cell lipid extraction, the same protocol was employed but with some modifications. Shortly, OMVs (4 mg, dry weight) were resuspended in 500 μl of 1x PBS and placed in a glass tube, to which 650 μl of chloroform and 1.3 ml of methanol were added. Samples were vigorously vortexed for 5 min, and then incubated at 30 °C, 160 rpm for 3 h. During the incubation, samples were vigorously vortexed for 1 min every 15 min. Afterwards, 650 μl of chloroform and 650 ml of MilliQ water were added, and samples were incubated for 30 min as before. To separate the organic lipid-rich phase from the aqueous phase, samples were centrifuged at 3,220*g* for 5 min at RT. The organic phase was then transferred to a glass vial, which was kept under a laminar flow hood overnight at RT to allow organic solvents to evaporate. The glass vials containing the dry pellets (lipid extracts) were stored at −20 °C. For whole-cell lipid extraction, bacterial cultures were grown as described in “Separation of the inner membrane and outer membrane”. Then, the equivalent to OD_600_:4.5 was harvested, washed in 1xPBS, and lipid were extracted as described above.

#### Lipid analysis by Thin Layer Chromatography (TLC).

Lipid extracts were first resuspended in 50 μl of chloroform:methanol (1:1) mixture, and then 15 μl (5 ml at a time) were spotted via a glass microsyringe onto a TLC aluminum plate (HPTLC Silica gel 60 F254 10 × 10 cm, MERCK). For lipid separation, a solvent system of chloroform: methanol:ammonium hydroxide (140:60:10, v/v/v) was used as previously described.^36^ To visualize lipids, the TLC plate was first placed in a glass jar saturated with iodine (I_2_) vapor from I_2_ crystals, exposed to for 30 min at RT, and then photographed. Afterwards, the TLC plate was kept at RT to allow I_2_ to decolorize. Then, the same TLC plate was soaked with ninhydrin (0.2% w/v in ethanol) for a few seconds, and then incubated at 150 °C for 10 min to develop spots. For 2D-TLC, lipids were separated using the same solvent system as above for the first dimension, whereas a solvent system consisting of chloroform:methanol:glacial acetic acid:acetone:water (130:10:10:20:3, v/v/v/v/v) was used for the second dimension. Lipids were visualized by spraying a solution of primuline (0.05% w/v in a water/acetone mixture (4:1)). To develop spots, plates exposed to UV-light (364 nm). For the relative quantification of each lipid class ImageJ was used.

#### Lipidomic analysis by LC-MS.

Lipid extracts were dried out under a laminar flow and then analyzed by LC-MS. Normal phase (NP) LC was performed on an Agilent 1200 Quaternary LC system equipped with an Ascentis Silica HPLC column, 5 μm, 25 cm × 2.1 mm (Sigma-Aldrich, St. Louis, MO) as previously described.^79^ Mobile phase A was a mixture of chloroform/methanol/aqueous ammonium hydroxide (800:195:5, v/v/v); mobile phase B was a mixture of chloroform/metha nol/water/aqueous ammonium hydroxide (600:340:50:5, v/v/v); mobile phase C was a mixture of chloroform/methanol/water/aqueous ammonium hydroxide (450:450:95:5, v/v/v). The elution program was as follows: 100% mobile phase A was held isocratically for 2 min and then linearly increased to 100% mobile phase B over 14 min and held at 100% B for 11 min. The LC gradient was then changed to 100% mobile phase C over 3 min and maintained at 100% C for 3 min, and finally returned to 100% A over 0.5 min and maintained at 100% A for 5 min. The LC eluent (with a total flow rate of 300 μL/min) was introduced into the ESI source of a high-resolution TripleTOF5600 mass spectrometer (Sciex, Framingham, MA). The instrumental settings for negative ion ESI and MS/MS analysis were as follows: IS = −4500 V; CUR = 20 psi; GSI = 20 psi; DP = −55 V; and FP = −150 V. For MS/MS analysis nitrogen was used as the collision gas. Analyst TF1.5 software (Sciex, Framingham, MA) was used for data analysis.

### LPS silver staining by AgNO_3_

#### Sample preparation.

For the assessment of LPS content in whole-cell lysates, bacterial cells were grown as described in “Separation of the inner membrane and outer membrane”. Samples, collected after 12 h of growth, were normalized to OD_600_:1 in 1 ml of 1x PBS. Then, cells from 750 μl of this bacterial suspension were harvested at 5,000*g* for 5 min and resuspended in 125 μl of 1x Laemmli buffer. Samples were then boiled at 99 °C for 10 min. Afterward, proteinase K was added (50 μg/ml final concentration), and samples were incubated at 37 °C overnight. Samples were boiled again at 99 °C for 10 min, and a second volume of proteinase K (equal to the first one) was then added. Samples were incubated at 55 °C for 3 h and boiled thereafter at 99 °C for 5 min before being loaded onto a SDS-polyacrylamide gel (15%) for SDS-PAGE.

For LPS detection in the membrane fractions and OMVs, the same protocol as above was carried out but using 7 mg (dried weight) of samples.

#### Staining.

After gel electrophoresis, LPS was visualized following a previously described protocol.^80^ Shortly, the SDS-polyacrylamide gel was fixed overnight in 50 ml of fixing solution consisting of ethanol:acetic acid:water (40:5:55, v/v/v) and then exposed to 50 ml of periodic acid solution (0.7% periodic acid in fixing solution, w/v) for 7 min to oxidase LPS. Afterward, the gel was washed 3 times with water (15 min each time) before being incubated with the staining solution for 10 min at RT. The staining solution was made by adding 2 ml of 5 M NH_4_OH and 28 ml of 0.1 N NaOH to 115 ml of MilliQ water. Then, 2.5 ml of a 20% (w/v) AgNO_3_ solution was added dropwise to the stirred NH_4_OH-NaOH mixture. After LPS staining, the gel was washed 3 times with water (10 min each time) and the LPS bands were revealed by exposing the gel to a solution of citric acid (10 mg) and formaldehyde (37%, 0.1 ml) dissolved in 200 ml of water. Upon band revelation, the gel was washed several times with water and finally photographed.

### In vivo crosslinking and TamL pull-down assays

*F. johnsoniae* (3xFLAG)*tamL*/(2xStrep)*tamB* was used to pull-down TamL, while *F. johnsoniae* (2xStrep)TamB was used as mock (SI Appendix, Table S6). From overnight pre-cultures, strains were inoculated in CYE (200 ml) at 30 °C, 160 rpm. When OD_600_ reached 0.6–0.8 (within 4–5 h), bacterial cultures were first harvested at 5,000*g* at 4 °C for 20 min, washed in 25 ml of 1x PBS, and harvested again. Pellets were resuspended in 10 ml of 1x PBS, and 3,3′-dithio bis(sulfosuccinimidyl propionate) (DTSSP, Sigma-Aldrich) at the final concentration of 1 mM was added for *in vivo* crosslinking. The DTSSP-treated cultures were incubated at 30 °C for 30 min at 125 rpm. Then, a solution of Tris-HCl, pH 7.5 (final concentration 100 mM) was added to quench DTSSP, and cultures were left at 30 °C for 30 min at 125 rpm. Cultures were then harvested and washed in 1x PBS as above. Pellets were stored overnight at −80 °C. The day after, pellets were resuspended on ice in 20 mM Tris-HCl, pH 7.5, 150 mM NaCl, 1 mM EDTA, lysozyme (0.4 mg/ml), DNase-I (30 μg/ml), 1% n-Dodecyl-β-D-Maltoside (DDM, ThermoFisher Scientific^™^), half tablet of EDTA-free protease inhibitors. Cells were lysed through a cell disruptor (Constant Systems) at 35,000 psi, and then centrifuged twice at 2,700*g* at 4 °C for 10 min. Supernatants were diluted in 1.5x their volume in 10 mM Tris-HCl, pH 7.5, 150 mM NaCl, 0.5 mM EDTA, 1% DDM. 25 μl of pre-washed FLAG-beads (DYKDDDDK Fab-Trap^™^ Agarose, ChromoTek) was added to each supernatant and incubated for 2 h at 4 °C on a tube roller under moderate agitation. Afterwards, supernatants were centrifuged at 2,500*g* at 4 °C for 10 min. Pellets, containing the FLAG-beads, were washed five times in 500 μl of 10 mM Tris-HCl, pH 7.5, 150 mM NaCl, 0.5 mM EDTA, 0.005% DDM. Crosslinked (3xFLAG)TamL was then eluted three times in 50 μl of 200 mM glycine, pH 2.5, and the pH was then neutralized. The total protein content of the eluates was determined by Pierce^™^ BCA Protein Assay (ThermoFisher Scientific^™^) and the presence of 3xFLAG-tagged TamL in the eluates was assessed by immunoblot. In parallel, eluate purity was determined by resolving 2 μg of total proteins onto a polyacrylamide gel (12%) followed by AgNO_3_ staining.^74^ The eluates with the highest quantity of (3xFLAG)TamL and protein purity when confronted with their mocks were pooled and analyzed by MS.

### Immunoblot analysis

A culture volume corresponding to OD_600_:1 was withdrawn and centrifuged at 5,000*g* for 5 min at RT. The resulting pellet was resuspended in 100 μl of 1x Laemmli sample buffer, boiled at 99 °C for 5 min, and centrifuged at high speed (17,000*g*) for 3 min. 10 μl was loaded onto SDS-polyacrylamide gels (8%) for electrophoresis. Proteins were then transferred onto a nitrocellulose membrane utilizing a semi-dry transfer cell (Bio-rad). For (3xFLAG)TamL and GroEL detection, blocking was carried out overnight at 4 °C in 5% (w/v) non-fat dry milk in phosphate buffer saline (PBS) with 0.05% (w/v) Tween 20 (PBST), and membranes were probed with anti-FLAG M2 (1:5,000; Sigma-Aldrich), StrepMAB-Classic (1:2,500; IBA-Lifesciences), or anti-GroEL (1:160,000; Sigma-Aldrich), for 3 and 1 h, respectively, at RT. For (2xStrep)TamB detection, blocking was performed for 1 h at RT in 5% (w/v) non-fat dry milk in PBST, and then membranes were probed with StrepMAB-Classic as described above overnight at 4 °C. Thereafter, membranes were immunoblotted for 1 h with secondary antibodies (1:5,000) anti-mouse or antirabbit linked to peroxidase (Dako Agilent). Signal for (3xFLAG)TamL was generated using Clarity^™^ Western ECL substrate chemiluminescence reagent (Bio-rad), while signal for GroEL and (2xStrep)TamB was obtained using KPL LumiGLO Reserve Chemiluminescent Substrate Kit (SeraCare). An Amersham Imager 600 (GE Healthcare) was used for signal detection.

### Microscopy techniques

#### Phase-contrast microscopy.

At the specified time points, cells grown in CYE (±1 mM IPTG) were first diluted to OD_600_:1, and then 100 μl was fixed in the presence of 300 μl of 4% (w/v) PFA for 15 min at RT. Afterwards, fixed cells were harvested at 5,000*g* for 5 min at RT, then resuspended in 100 μl of sterile 1x PBS, and stored at 4 °C. When needed, 3 μl of fixed bacteria was spotted on 1% agarose PBS pads, and pictures were taken using an Axioscop (Zeiss) microscope with an Orca-Flash 4.0 camera (Hamamatsu) and Zen Softmax Pro software (Zeiss).

#### Transmission Electron Microscopy (TEM) of F. johnsoniae cells.

An equivalent of OD_600_:3 was harvested using the same protocol as the membrane fractionation experiments. Samples were then prepared following an already described protocol.^81^ Ultrathin sections were cut with a DiATOME ultra 45° diamond knife, deposited into a copper grid and then viewed on a TECNAi 10 transmission electron microscope (PHILIPS) equipped with a megaview CCD camera of 1024x1024 pixel resolution (Olympus) operating at an acceleration voltage of 80 kV. Images were taken via Soft Imaging System (Olympus).

#### Negative staining of OMVs for TEM.

A previously described protocol was used,^82^ though with some modifications. Shortly, carbon-coated copper grids for TEM were negatively charged and hydrophilized using a Q150T S/E/ES device (Quorum Technologies) following the manufacturer’s instructions. The day after, 3 μl of bacterial OMVs were deposited onto a negatively charged and hydrophilized grid, incubated at RT for 3 min and then quickly wash with MilliQ water. The grid was then deposited for 3 min at RT onto 1% aqueous uranyl acetate (Sigma-Aldrich). Excess liquid was gently removed, and grids were allowed to air dry. Samples were views as described above. A total of *n* = 102 OMVs from three independent experiments were photographed and their diameter was measured by ImageJ.

### AlphaFold2 structural predictions

The structural models were retrieved from the AlphaFold Protein Structure Database (https://alphafold.ebi.ac.uk/). For TamB and TamB2 from *F. johnsoniae* and TamB from *C. canimorsus*, the structures were generated using AlphaFold2.^83^ The full-length structures of (2xStrep)TamB and (3xFLAG)TamL from *F. johnsoniae*, were instead obtained via the AlphaFold2 tool available elsewhere.^84^ From the same platform, the AlphaFold Multimer tool was also used to predict TamL-TamB complex. The best-scored models were used for analysis and displayed in a cartoon representation using PyMOL Molecular Graphics Systems Version 2.5.2 (Schrödinger, LCC).

### Conservation of TamL and TamB proteins in Bacteroidota

A previously published protocol was used.^49^ Briefly, A DELTA-BLAST sequence similarity search was conducted on the 30 Bacteroidota species using the TamA, −L, −B protein sequences of *F. johnsoniae* ATCC 17061 UW101 as queries: TamA (WP_012022494.1), TamL (WP_012023543.1), TamB (WP_012026557.1), TamL2 (WP_012023976.1) and TamB2 (WP_012023975.1). The genomic neighborhood of all hits with an E value ≤0.001 was inspected using MaGe.^85^ The structure of the corresponding proteins was predicted using AlphaFold server (with default parameters)^86^ and compared with the corresponding structures of the of *F. johnsoniae* initial queries by manual inspection. The phylogenetic tree based on NCBI taxonomy was generated via phyloT.^87^

## Supplementary Material

Supp material

## Data Availability

The mass spectrometry proteomics data are publicly available in the ProteomeXchange Consortium via the PRIDE^88^ partner repository with the dataset identifier PXD056806 and https://doi.org/10.6019/PXD056806.

## References

[1] Silhavy TJ, Kahne D, Walker S, (2010). The bacterial cell envelope. Cold Spring Harb. Perspect. Biol 2, a000414. 10.1101/cshperspect.a000414. 20452953 PMC2857177

[2] Guest RL, Silhavy TJ, (2023). Cracking outer membrane biogenesis. Biochim. Biophys. Acta – Mol. Cell Res 1870, 119405. 10.1016/j.bbamcr.2022.119405. 36455781 PMC9878550

[3] Okuda S, Tokuda H, (2011). Lipoprotein sorting in bacteria. Annu. Rev. Microbiol 65, 239–259. 10.1146/annurev-micro-090110-102859. 21663440

[4] Gatsos X, Perry AJ, Anwari K, Dolezal P, Wolynec PP, Likić VA, Purcell AW, Buchanan SK, Lithgow T, (2008). Protein secretion and outer membrane assembly in Alphaproteobacteria. FEMS Microbiol. Rev 32, 995–1009. 10.1111/j.1574-6976.2008.00130.x. 18759741 PMC2635482

[5] Simmerman RF, Dave AM, Bruce BD (2014). Structure and Function of POTRA Domains of Omp85/TPS Superfamily, Elsevier Inc. 10.1016/B978-0-12-800097-7.00001-4. 24411168

[6] Wu T, Malinverni J, Ruiz N, Kim S, Silhavy TJ, Kahne D, (2005). Identification of a multicomponent complex required for outer membrane biogenesis in Escherichia coli. Cell 121, 235–245. 10.1016/j.cell.2005.02.015. 15851030

[7] Voulhoux R, Bos MP, Geurtsen J, Mols M, Tommassen J, (2003). Role of a highly conserved bacterial protein in outer membrane protein assembly. Science (80-.) 299, 262–265. 10.1126/science.1078973. 12522254

[8] Genevrois S, Steeghs L, Roholl P, Letesson JJ, Van der Ley P, (2003). The Omp85 protein of Neisseria meningitidis is required for lipid export to the outer membrane. EMBO J. 22, 1780–1789. 10.1093/emboj/cdg174. 12682011 PMC154466

[9] Heinz E, Lithgow T, (2014). A comprehensive analysis of the Omp85/TpsB protein superfamily structural diversity, taxonomic occurrence, and evolution. Front. Microbiol 5, 1–13. 10.3389/fmicb.2014.00370. 25101071 PMC4104836

[10] Goh KJ, Stubenrauch CJ, Lithgow T, (2024). The TAM, a Translocation and Assembly Module for protein assembly and potential conduit for phospholipid transfer. EMBO Rep. 25, 1711–1720. 10.1038/s44319-024-00111-y. 38467907 PMC11014939

[11] Stubenrauch CJ, Lithgow T, (2019). The TAM: a translocation and assembly module of the β-barrel assembly machinery in bacterial outer membranes. EcoSal Plus 8, 1–7. 10.1128/ecosalplus.esp-0036-2018. PMC11573294 30816086

[12] Albenne C, Ieva R, (2017). Job contenders: roles of the β-barrel assembly machinery and the translocation and assembly module in autotransporter secretion. Mol. Microbiol 106, 505–517. 10.1111/mmi.13832. 28887826

[13] Wang X, Nyenhuis SB, Bernstein HD, (2024). The translocation assembly module (TAM) catalyzes the assembly of bacterial outer membrane proteins *in vitro*. Nature Commun. 15, 1–15. 10.1038/s41467-024-51628-8. 39174534 PMC11341756

[14] Selkrig J, Mosbahi K, Webb CT, Belousoff MJ, Perry AJ, Wells TJ, Morris F, Leyton DL, Totsika M, Phan MD, Celik N, Kelly M, Oates C, Hartland EL, Robins-Browne RM, Ramarathinam SH, Purcell AW, Schembri MA, Strugnell RA, Henderson IR, Walker D, Lithgow T, (2012). Discovery of an archetypal protein transport system in bacterial outer membranes. Nature Struct. Mol. Biol 19, 506–510. 10.1038/nsmb.2261. 22466966

[15] Selkrig J, Belousoff MJ, Headey SJ, Heinz E, Shiota T, Shen HH, Beckham SA, Bamert RS, Phan MD, Schembri MA, Wilce MCJ, Scanlon MJ, Strugnell RA, Lithgow T, (2015). Conserved features in TamA enable interaction with TamB to drive the activity of the translocation and assembly module. Sci. Rep 5, 1–12. 10.1038/srep12905. PMC4525385 26243377

[16] Gruss F, Zähringer F, Jakob RP, Burmann BM, Hiller S, Maier T, (2013). The structural basis of autotransporter translocation by TamA. Nature Struct. Mol. Biol 20, 1318–1320. 10.1038/nsmb.2689. 24056943

[17] Mellouk A, Jaouen P, Ruel L-J, Lê M, Martini C, Moraes TF, El Bakkouri M, Lagüe P, Boisselier E, Calmettes C, (2024). POTRA domains of the TamA insertase interact with the outer membrane and modulate membrane properties. PNAS 121, e2402543121. 10.1073/pnas.2402543121. 38959031 PMC11252910

[18] Josts I, Stubenrauch CJ, Vadlamani G, Mosbahi K, Walker D, Lithgow T, Grinter R, (2017). The structure of a conserved domain of TamB reveals a hydrophobic β taco fold. Structure 25, 1898–1906.e5. 10.1016/j.str.2017.10.002. 29129383 PMC5719984

[19] Mcdonnell RT, Patel N, Wehrspan ZJ, Elcock AH, (2023). Atomic models of all major trans-envelope complexes involved in lipid trafficking in *Escherichia coli* constructed using a combination of AlphaFold2, AF2Complex, and membrane morphing simulations keywords. BioRxiv [preprint]. 10.1101/2023.04.28.538765.

[20] Kumar S, Ruiz N, (2023). Bacterial AsmA-like proteins: bridging the gap in intermembrane phospholipid transport. Contact 6, 1–9. 10.1177/25152564231185931. PMC10345924 37455811

[21] Sposato D, Mercolino J, Torrini L, Sperandeo P, Lucidi M, Alegiani R, Varone I, Molesini G, Leoni L, Rampioni G, Visca P, Imperi F, (2024). Redundant essentiality of AsmA-like proteins in *Pseudomonas aeruginosa*. Msphere 9, e00677–23. 10.1128/msphere.00677-23. 38305166 PMC10900882

[22] Neuman SD, Levine TP, Bashirullah A, (2022). A novel superfamily of bridge-like lipid transfer proteins. Trends Cell Biol. 32, 962–974. 10.1016/j.tcb.2022.03.011. 35491307 PMC9588498

[23] Melia TJ, Reinisch KM, (2022). A possible role for VPS13-family proteins in bulk lipid transfer, membrane expansion and organelle biogenesis. J. Cell Sci 135 10.1242/jcs.259357. PMC8976877 35267021

[24] McEwan DG, Ryan KM, (2022). ATG2 and VPS13 proteins: molecular highways transporting lipids to drive membrane expansion and organelle communication. FEBS J. 289, 7113–7127. 10.1111/febs.16280. 34783437

[25] Ruiz N, Davis RM, Kumar S, (2021). YhdP, TamB, and YdbH are redundant but essential for growth and lipid homeostasis of the gram-negative outer membrane. MBio 12 10.1128/mBio.02714-21. PMC8593681 34781743

[26] Douglass MV, McLean AB, Trent MS, (2022). Absence of YhdP, TamB, and YdbH leads to defects in glycerophospholipid transport and cell morphology in gram-negative bacteria. PLoS Genet. 18, 1–21. 10.1371/journal.pgen.1010096. PMC8912898 35226662

[27] Cooper BF, Clark R, Kudhail A, Dunn D, Tian Q, Bhabha G, Ekiert DC, Khalid S, Isom GL, (2024). Phospholipid transport across the bacterial periplasm through the envelope-spanning bridge YhdP. J. Mol. Biol 168891. 10.1016/j.jmb.2024.168891. 39638236

[28] Rai AK, Sawasato K, Bennett HC, Kozlova A, Sparagna GC, Bogdanov M, Mitchell AM, (2024). Genetic evidence for functional diversification of gram-negative intermembrane phospholipid transporters. PLoS Genet. 20, 1–26. 10.1371/journal.pgen.1011335. PMC11226057 38913742

[29] Kumar S, Davis RM, Ruiz N, (2024). YdbH and YnbE form an intermembrane bridge to maintain lipid homeostasis in the outer membrane of *Escherichia coli*. PNAS 121 10.1073/pnas.2321512121. PMC11126948 38748582

[30] Heinz E, Selkrig J, Belousoff MJ, Lithgow T, (2015). Evolution of the translocation and assembly module (TAM). Genome Biol. Evol 7, 1628–1643. 10.1093/gbe/evv097. 25994932 PMC4494059

[31] Iqbal H, Kenedy MR, Lybecker M, Akins DR, (2016). The TamB ortholog of Borrelia burgdorferi interacts with the β-barrel assembly machine (BAM) complex protein BamA. Mol. Microbiol 102, 757–774. 10.1111/mmi.13492. 27588694 PMC5582053

[32] Foley MH, Cockburn DW, Koropatkin NM, (2016). The Sus operon: a model system for starch uptake by the human gut Bacteroidetes. Cell. Mol. Life Sci 73, 2603–2617. 10.1007/s00018-016-2242-x. 27137179 PMC4924478

[33] Manfredi P, Renzi F, Mally M, Sauteur L, Schmaler M, Moes S, Jenö P, Cornelis GR, (2011). The genome and surface proteome of *Capnocytophaga canimorsus* reveal a key role of glycan foraging systems in host glycoproteins deglycosylation. Mol. Microbiol 81, 1050–1060. 10.1111/j.1365-2958.2011.07750.x. 21762219

[34] Lauber F, Cornelis GR, Renzi F, (2016). Identification of a new lipoprotein export signal in gram-negative bacteria. MBio 7 10.1128/mBio.01232-16. PMC5080379 27795390

[35] Pitta TP, Leadbetter ER, Godchaux W, (1989). Increase of ornithine amino lipid content in a sulfonolipid-deficient mutant of *Cytophaga johnsonae*. J. Bacteriol 171, 952–957. 10.1128/jb.171.2.952-957.1989. 2914878 PMC209687

[36] Vences-Guzmán MÁ, Peña-Miller R, Hidalgo-Aguilar NA, Vences-Guzmán ML, Guan Z, Sohlenkamp C, (2021). Identification of the *Flavobacterium johnsoniae* cysteate-fatty acyl transferase required for capnine synthesis and for efficient gliding motility. Environ. Microbiol 23, 2448–2460. 10.1111/1462-2920.15445. 33626217 PMC9364336

[37] McBride MJ, Xie G, Martens EC, Lapidus A, Henrissat B, Rhodes RG, Goltsman E, Wang W, Xu J, Hunnicutt DW, Staroscik AM, Hoover TR, Cheng YQ, Stein JL, (2009). Novel features of the polysaccharide-digesting gliding bacterium *Flavobacterium johnsoniae* as revealed by genome sequence analysis. Appl. Environ. Microbiol 75, 6864–6875. 10.1128/AEM.01495-09. 19717629 PMC2772454

[38] McBride MJ, (2019). Bacteroidetes gliding motility and the type IX secretion system. Protein Secret. Bact 7, 363–374. 10.1128/9781683670285.ch29. PMC11588200 30767845

[39] Johnston JJ, Shrivastava A, McBride MJ, (2017). Untangling *Flavobacterium johnsoniae* gliding motility and protein secretion. J Bacteriol 20, 2. 10.1128/JB.00362-17 PMC5738736 29109184

[40] Mally M, Paroz C, Shin H, Meyer S, Soussoula LV, Schmiediger U, Saillen-Paroz C, Cornelis GR, (2009). Prevalence of *Capnocytophaga canimorsus* in dogs and occurrence of potential virulence factors. Microbes Infect. 11, 509–514. 10.1016/j.micinf.2009.02.005. 19285152

[41] Butler T., (2015). *Capnocytophaga canimorsus*: an emerging cause of sepsis, meningitis, and post-splenectomy infection after dog bites. Eur. J. Clin. Microbiol. Infect. Dis. off. Publ. Eur. Soc. Clin. Microbiol 34, 1271–1280. 10.1007/s10096-015-2360-7. 25828064

[42] Boratyn GM, Schäffer AA, Agarwala R, Altschul SF, Lipman DJ, Madden TL, (2012). Domain enhanced lookup time accelerated BLAST. Biol. Direct 7, 1–14. 10.1186/1745-6150-7-12. 22510480 PMC3438057

[43] Teufel F, Almagro Armenteros JJ, Johansen AR, G´ıslason MH, Pihl SI, Tsirigos KD, Winther O, Brunak, von Heijne G, Nielsen H, (2022). SignalP 6.0 predicts all five types of signal peptides using protein language models. Nature Biotechnol. 40, 1023–1025. 10.1038/s41587-021-01156-3. 34980915 PMC9287161

[44] Grimm J, Shi H, Wang W, Mitchell AM, Wingreen NS, Huang KC, Silhavy TJ, (2020). The inner membrane protein YhdP modulates the rate of anterograde phospholipid flow in *Escherichia coli*. PNAS 117, 26907–26914. 10.1073/pnas.2015556117. 33046656 PMC7604412

[45] Sutterlin HA, Shi H, May KL, Miguel A, Khare S, Huang KC, Silhavy TJ, (2016). Disruption of lipid homeostasis in the Gram-negative cell envelope activates a novel cell death pathway. PNAS 113, E1565–E1574. 10.1073/pnas.1601375113. 26929379 PMC4801249

[46] Sato K, Naito M, Yukitake H, Hirakawa H, Shoji M, McBride MJ, Rhodes RG, Nakayama K, (2010). A protein secretion system linked to bacteroidete gliding motility and pathogenesis. PNAS 107, 276–281. 10.1073/pnas.0912010107. 19966289 PMC2806738

[47] Tan Z, Black W, Yoon JM, Shanks JV, Jarboe LR, (2017). Improving *Escherichia coli* membrane integrity and fatty acid production by expression tuning of FadL and OmpF. Microb. Cell Fact 16, 1–15. 10.1186/s12934-017-0650-8. 28245829 PMC5331629

[48] McBride MJ, Nakane D, (2015). Flavobacterium gliding motility and the type IX secretion system. Curr. Opin. Microbiol 28, 72–77. 10.1016/j.mib.2015.07.016. 26461123

[49] De Smet T, Baland E, Giovannercole F, Mignon J, Lizen L, Dugauquier R, Lauber F, Dieu M, Lima-Mendez G, Michaux C, Devos D and Renzi F, LolA and LolB are conserved in Bacteroidota and are crucial for gliding motility and Type IX secretion, Commun. Bio., 8:376, 2025, 10.1038/s42003-025-07817-2. PMC11885536 40050408

[50] Rhodes RG, Samarasam MN, Van Groll EJ, McBride MJ, (2011). Mutations in *Flavobacterium johnsoniae* sprE result in defects in gliding motility and protein secretion. J. Bacteriol 193, 5322–5327. 10.1128/JB.05480-11. 21784937 PMC3187464

[51] Grondin JM, Tamura K, Déjean G, Abbott DW, Brumer H, (2017). Polysaccharide utilization loci: Fueling microbial communities. J. Bacteriol 199 10.1128/JB.00860-16. PMC5512228 28138099

[52] Vences-Guzmán MÁ, Guan Z, Escobedo-Hinojosa WI, Bermúdez-Barrientos JR, Geiger O, Sohlenkamp C, (2015). Discovery of a bifunctional acyltransferase responsible for ornithine lipid synthesis in *Serratia proteamaculans*. Environ. Microbiol 17, 1487–1496. 10.1111/1462-2920.12562. 25040623

[53] Evans R, O’Neill M, Pritzel A, Antropova N, Senior A, Green T, Žídek A, Bates R, Blackwell S, Yim J, Ronneberger O, Bodenstein S, Zielinski M, Bridgland A, Potapenko A, Cowie A, Tunyasuvunakool K, Jain R, Clancy E, Kohli P, Jumper J, Hassabis D, (2022). Protein complex prediction with AlphaFold-multimer. BioRxiv2021.10.04.463034. 10.1101/2021.10.04.463034.

[54] Gu Y, Li H, Dong H, Zeng Y, Zhang Z, Paterson NG, Stansfeld PJ, Wang Z, Zhang Y, Wang W, Dong C, (2016). Structural basis of outer membrane protein insertion by the BAM complex. Nature 531, 64–69. 10.1038/nature17199. 26901871

[55] Tomasek D, Rawson S, Lee J, Wzorek JS, Harrison SC, Li Z, Kahne D, (2020). Structure of a nascent membrane protein as it folds on the BAM complex. Nature 583, 473–478. 10.1038/s41586-020-2370-1. 32528179 PMC7367713

[56] Shen C, Chang S, Luo Q, Chan KC, Zhang Z, Luo B, Xie T, Lu G, Zhu X, Wei X, Dong C, Zhou R, Zhang X, Tang X, Dong H, (2023). Structural basis of BAM-mediated outer membrane β-barrel protein assembly. Nature 617, 185–193. 10.1038/s41586-023-05988-8. 37100902

[57] Shoji M, Shibata S, Sueyoshi T, Naito M, Nakayama K, (2020). Biogenesis of Type V pili. Microbiol. Immunol 64, 643–656. 10.1111/1348-0421.12838. 32816331

[58] Bialer MG, Ruiz-Ranwez V, Sycz G, Estein SM, Russo DM, Altabe S, Sieira R, Zorreguieta A, (2019). MapB, the Brucella suis TamB homologue, is involved in cell envelope biogenesis, cell division and virulence. Sci. Rep 9, 1–18. 10.1038/s41598-018-37668-3. 30770847 PMC6377625

[59] Martorana AM, Motta S, Di Silvestre D, Falchi F, Dehò G, Mauri P, Sperandeo P, Polissi A, (2014). Dissecting *Escherichia coli* outer membrane biogenesis using differential proteomics. PLoS One 9 10.1371/journal.pone.0100941. PMC4072712 24967819

[60] Asmar AT, Collet JF, (2018). Lpp, the Braun lipoprotein, turns 50—major achievements and remaining issues. FEMS Microbiol. Lett 365, 1–8. 10.1093/femsle/fny199. 30107563

[61] Liao CT, Li CE, Chang HC, Hsu CH, Chiang YC, Hsiao YM, (2022). The lolB gene in *Xanthomonas campestris* pv. campestris is required for bacterial attachment, stress tolerance, and virulence. BMC Microbiol. 22, 1–13. 10.1186/s12866-021-02416-7. 34996353 PMC8739992

[62] Malinverni JC, Werner J, Kim S, Sklar JG, Kahne D, Misra R, Silhavy TJ, (2006). YfiO stabilizes the YaeT complex and is essential for outer membrane protein assembly in *Escherichia coli*. Mol. Microbiol 61, 151–164. 10.1111/j.1365-2958.2006.05211.x. 16824102

[63] Mikheyeva IV, Sun J, Huang KC, Silhavy TJ, (2023). Mechanism of outer membrane destabilization by global reduction of protein content. Nature Commun. 14, 1–9. 10.1038/s41467-023-40396-6. 37714857 PMC10504340

[64] Werner J, Misra R, (2005). YaeT (Omp85) affects the assembly of lipid-dependent and lipid-independent outer membrane proteins of *Escherichia coli*. Mol. Microbiol 57, 1450–1459. 10.1111/j.1365-2958.2005.04775.x. 16102012

[65] Sartorio MG, Pardue EJ, Scott NE, Feldman MF, (2023). Human gut bacteria tailor extracellular vesicle cargo for the breakdown of diet- and host-derived glycans. PNAS 120, e2306314120. 10.1073/pnas.2306314120. 37364113 PMC10319031

[66] Valguarnera E, Scott NE, Azimzadeh P, Feldman MF, (2018). Surface exposure and packing of lipoproteins into outer membrane vesicles are coupled processes in bacteroides. Msphere 3, 1–14. 10.1128/msphere.00559-18. PMC6222051 30404931

[67] McBride MJ, Kempf MJ, (1996). Development of techniques for the genetic manipulation of the gliding bacterium *Cytophaga johnsonae*. J. Bacteriol 178, 583–590. 10.1128/jb.178.3.583-590.1996. 8550486 PMC177698

[68] Jun L, J. MM, Sriram S, (2007). Cell surface filaments of the gliding bacterium *Flavobacterium johnsoniae* revealed by cryo-electron tomography. J. Bacteriol 189, 7503–7506. 10.1128/jb.00957-07. 17693495 PMC2168446

[69] Gibson DG, Young L, Chuang RY, Venter JC, Hutchison CA, Smith HO, (2009). Enzymatic assembly of DNA molecules up to several hundred kilobases. Nature Methods 6, 343–345. 10.1038/nmeth.1318. 19363495

[70] Parker AC, Smith CJ, (2012). Development of an IPTG inducible expression vector adapted for *Bacteroides fragilis*. Plasmid 68, 86–92. 10.1007/978-981-99-9283-6_2167. 22487080 PMC3389198

[71] Dumetz F, Duchaud E, Claverol S, Orieux N, Papillon S, Lapaillerie D, Le Hénaff M, (2008). Analysis of the *Flavobacterium psychrophilum* outer-membrane subproteome and identification of new antigenic targets for vaccine by immunomics. Microbiology 154, 1793–1801. 10.1099/mic.0.2008/016600-0. 18524934

[72] Hunnicutt DW, McBride MJ, (2000). Cloning and characterization of the *Flavobacterium johnsoniae* gliding-motility genes gldB and gldC. J. Bacteriol 182, 911–918. 10.1128/JB.182.4.911-918.2000. 10648514 PMC94364

[73] Kasahara M, Anraku Y, (1974). Succinate dehydrogenase of *Escherichia coli* membrane vesicles. Activation and properties of the enzyme. J. Biochem 76, 959–966. 4616033

[74] Rabilloud T, Carpentier G, Tarroux P, (1988). Improvement and simplification of low-background silver staining of proteins by using sodium dithionite. Electrophoresis 9, 288–291. 10.1002/elps.1150090608. 2466660

[75] Meier F, Brunner A-D, Koch S, Koch H, Lubeck M, Krause M, Goedecke N, Decker J, Kosinski T, Park MA, Bache N, Hoerning O, Cox J, Räther O, Mann M, (2018). Online parallel accumulation-serial fragmentation (PASEF) with a novel trapped ion mobility mass spectrometer. Mol. Cell. Proteomics 17, 2534–2545. 10.1074/mcp.TIR118.000900. 30385480 PMC6283298

[76] Yu NY, Wagner JR, Laird MR, Melli G, Rey S, Lo R, Dao P, Cenk Sahinalp S, Ester M, Foster LJ, Brinkman FSL, (2010). PSORTb 3.0: Improved protein subcellular localization prediction with refined localization subcategories and predictive capabilities for all prokaryotes. Bioinformatics 26, 1608–1615. 10.1093/bioinformatics/btq249. 20472543 PMC2887053

[77] Drula E, Garron ML, Dogan S, Lombard V, Henrissat B, Terrapon N, (2022). The carbohydrate-active enzyme database: functions and literature. Nucleic Acids Res. 50, D571–D577. 10.1093/nar/gkab1045. 34850161 PMC8728194

[78] Bligh WJ, Dyer EG, (1959). Can. J. Biochem. Physiol 37 10.1139/o59-099 13671378

[79] Joyce LR, Manzer HS, da Mendonça CJ, Villarreal R, Nagao PE, Doran KS, Palmer KL, Guan Z, (2022). Identification of a novel cationic glycolipid in *Streptococcus agalactiae* that contributes to brain entry and meningitis. PLOS Biol. 20, e3001555. 10.1371/journal.pbio.3001555. 35180210 PMC8893666

[80] Tsai C-M, Frash CE, (1982). A sensitive silver stain for detecting lipopolysaccharides in polyacrylamide gels. Anal. Biochem 119, 115–119 https://ac.els-cdn.com/000326978290673X/1-s2.0-000326978290673X-main.pdf?_tid=bf086faa-13e2-4036-a390-82b83207b4a1&acdnat=1548773814_ffae24506c70807a1b7185ec355a3d5a. 6176137 10.1016/0003-2697(82)90673-x

[81] Wenzel M, Dekker MP, Wang B, Burggraaf MJ, Bitter W, van Weering JRT, Hamoen LW, (2021). A flat embedding method for transmission electron microscopy reveals an unknown mechanism of tetracycline. Commun. Biol 4, 306. 10.1038/s42003-021-01809-8. 33686188 PMC7940657

[82] Pardue EJ, Sartorio MG, Jana B, Scott NE, Beatty WL, Ortiz-Marquez JC, Van Opijnen T, Hsu F-F, Potter RF, Feldman MF, (2024). Dual membrane-spanning anti-sigma factors regulate vesiculation in *Bacteroides thetaiotaomicron*. PNAS 121, e2321910121. 10.1073/pnas.2321910121. 38422018 PMC10927553

[83] Mirdita M, Schütze K, Moriwaki Y, Heo L, Ovchinnikov S, Steinegger M, (2022). ColabFold: making protein folding accessible to all. Nature Methods 19, 679–682. 10.1038/s41592-022-01488-1. 35637307 PMC9184281

[84] Cianfrocco MA, Wong-Barnum M, Youn C, Wagner R, Leschziner A, (2017). COSMIC2: A Science Gateway for Cryo-Electron Microscopy Structure Determination. In: Pract. Exp. Adv. Res. Comput. 2017 Sustain. Success Impact Association for Computing Machinery, New York, NY, USA. 10.1145/3093338.3093390.

[85] Vallenet D, Labarre L, Rouy Z, Barbe V, Bocs S, Cruveiller S, Lajus A, Pascal G, Scarpelli C, Médigue C, (2006). MaGe: a microbial genome annotation system supported by synteny results. Nucleic Acids Res. 34, 53–65. 10.1093/nar/gkj406. 16407324 PMC1326237

[86] Jumper J, Evans R, Pritzel A, Green T, Figurnov M, Ronneberger O, Tunyasuvunakool K, Bates R, Žídek A, Potapenko A, Bridgland A, Meyer C, Kohl SAA, Ballard AJ, Cowie A, Romera-Paredes B, Nikolov S, Jain R, Adler J, Back T, Petersen S, Reiman D, Clancy E, Zielinski M, Steinegger M, Pacholska M, Berghammer T, Bodenstein S, Silver D, Vinyals O, Senior AW, Kavukcuoglu K, Kohli P, Hassabis D, (2021). Highly accurate protein structure prediction with AlphaFold. Nature 596, 583–589. 10.1038/s41586-021-03819-2. 34265844 PMC8371605

[87] Letunic I, Doerks T, Bork P, (2012). SMART 7: recent updates to the protein domain annotation resource. Nucleic Acids Res. 40, D302–D305. 10.1093/nar/gkr931. 22053084 PMC3245027

[88] Perez-Riverol Y, Bai J, Bandla C, García-Seisdedos D, Hewapathirana S, Kamatchinathan S, Kundu DJ, Prakash A, Frericks-Zipper A, Eisenacher M, Walzer M, Wang S, Brazma A, Vizcaíno JA, (2022). The PRIDE database resources in 2022: a hub for mass spectrometry-based proteomics evidences. Nucleic Acids Res. 50, D543–D552. 10.1093/nar/gkab1038. 34723319 PMC8728295

